# Multimorbidities and Overprescription of Proton Pump Inhibitors in Older Patients

**DOI:** 10.1371/journal.pone.0141779

**Published:** 2015-11-04

**Authors:** Anne Delcher, Sylvie Hily, Anne Sophie Boureau, Guillaume Chapelet, Gilles Berrut, Laure de Decker

**Affiliations:** 1 Department of Geriatrics, Saint-Nazaire Hospital, Saint-Nazaire, France; 2 Department of Geriatrics, Nantes University Hospital, Nantes, France; University Hospital Llandough, UNITED KINGDOM

## Abstract

**Objectives:**

To determine whether there is an association between overprescription of proton pump inhibitors (PPIs) and multimorbidities in older patients.

**Design:**

Multicenter prospective study.

**Setting:**

Acute geriatric medicine at the University Hospital of Nantes and the Hospital of Saint-Nazaire.

**Participants:**

Older patients aged 75 and over hospitalized in acute geriatric medicine.

**Measurements:**

Older patients in acute geriatric medicine who received proton pump inhibitors. Variables studied were individual multimorbidities, the burden of multimorbidity evaluated by the Cumulative Illness Rating Scale, age, sex, type of residence (living in nursing home or not), functional abilities (Lawton and Katz scales), nutritional status (Body Mass Index), and the type of concomitant medications (antiaggregant, corticosteroids’, or anticoagulants).

**Results:**

Overprescription of proton pump inhibitors was found in 73.9% older patients. In the full model, cardiac diseases (odds ratio [OR] = 4.17, p = 0.010), metabolic diseases (OR = 2.14, p = 0.042) and corticosteroids (OR = 5.39, p = 0.028) were significantly associated with overprescription of proton pump inhibitors. Esogastric diseases (OR = 0.49, p = 0.033) were negatively associated with overprescription of proton pump inhibitors.

**Conclusion:**

Cardiac diseases and metabolic diseases were significantly associated with overprescription of proton pump inhibitors.

## Introduction

Proton pump inhibitors (PPIs) are one of the most frequently prescribed classes of drugs in the world [1]. PPIs are the most effective drugs available to reduce gastric acid secretion [2]. PPIs are effective in the cure or prevention of peptic acid disorders and in the management of gastroesophageal reflux disease (GERD), esophagitis, gastric ulcers, bleeding peptic ulcers, eradication of *Helicobacter pylori*, dyspepsia, Zollinger–Ellison syndrome, and the prevention of gastrointestinal toxicity induced by non-steroidal anti-inflammatory drugs (NSAIDs) [3]. PPIs combine a high level of efficacy and ease of use [4]. PPIs have been increasingly prescribed for chronic use, particularly in the older populations [5].

Although these drugs were initially considered safe, recent pharmacoepidemiological studies have shown an association between PPI use and long-term adverse effects [6]. Studies have shown an increase in the incidence of bone fractures (hip fractures and spine fractures [7]) and community-acquired pneumonia [8]. There seems to be an association between PPIs and *Clostridium difficile* infection [9]. Additionally, the available data are still insufficient to estimate the risk of deficiency in vitamin B12, iron, magnesium, or the risk of gastric cancer [10].

However, studies have shown that PPIs are not always prescribed with a clear indication [11]. Indeed, between 25% and 86% of older individuals taking a PPI have been overprescribed these medications [4, 5]. Numerous reasons for this overprescribing have been identified, such as the absence of treatment reevaluation, or the use of a antiaggregant [12]. Overprescribing has many outcomes, particularly in older people [3]. Indeed, in older patients, overprescribing of a PPI is associated with increases in morbidity, adverse drug events, hospitalization, and mortality [13]. One explanation for these outcomes is that older patients have a higher risk of adverse drug reactions, because they have multiple multimorbidities and may be taking numerous drugs [3, 11].

The multimorbidity can be assessed individually or by the burden of multimorbidities. The Charlson Comorbidity Index (CCI) and the Cumulative Illness Rating Scale (CIRS) are valid and reliable methods used in clinical research to measure the burden of multimorbidities [14]; however, the association between the overprescribing of PPIs and the burden of multimorbidities evaluated by the CCI or the CIRS has been inconsistent [5, 6, 12]. To our knowledge, no study evaluating the association between the individual multimorbidities and the overprescribing of PPIs has been performed.

Our hypothesis is that overprescription of PPIs is associated with the individual multimorbidities in older patients evaluated using the multimorbidities group criteria included in the CIRS. The aim of the present study was to establish a relationship between overprescribing of PPIs and multimorbidities in older patients.

## Methods

### Participants

This study was conducted from January 2014 to July 2014 in the acute geriatric care units in the University Hospital of Nantes and the Hospital of Saint-Nazaire, France. All inpatients in the acute geriatric care units aged 75 and older and treated with a PPI were eligible for this prospective observational study. The exclusion criteria were the patients whose prescription for a PPI had been introduced during an emergency and patients who either refused to participate in the study or did not have the capacity to give their consent.

### Clinical assessment

The endpoint of this study was the overprescription of a PPI. The overprescription was defined by the prescription of IPP not based on the recommendations issued by the french regulatory agency for the safety of health products (AFFSAPS) beginning in November 2007. AFFSAPS guarantees, through its mission, the sanitary safety, efficiency, quality, and good use of all healthcare products [15]. According to these recommendations, a PPI has four main indications: the treatment of GERD and esophagitis caused by GERD, the prevention and treatment of the gastroduodenal damage due to the non-steroidal anti-inflammatory drugs in patients at risk, the eradication of *H*. *pylori*, and the treatment of gastroduodenal ulcers.

The main variable studied was comprised of the individual multimorbidities, using the multimorbidities group criteria included in the CIRS. The multimorbidities groups studied were cardiac diseases, vascular diseases, respiratory diseases, eye and throat diseases, esogastric diseases, hepatic and renal diseases, urologic diseases, musculoskeletal/integumentary diseases, neurological diseases, metabolic diseases, and psychiatric diseases.

The burden of multimorbidities was assessed using the CIRS. The CIRS score is significantly associated with mortality, urgent hospitalizations, the number of drugs, biological anomalies, and the level of functional dependence [16].

Other variables were collected, including the age, sex, type of residence (living in a nursing home or not), functional abilities that were assessed using the Katz activity of daily living (ADL), and nutritional status that was assessed using the weight and Body Mass Index (BMI). The number of concomitant medications and the type of medications, such as antiaggregants, corticostéroids’, and anticoagulants were also collected. Other variables studied were whether the patients were taking a full dose PPI or not, and other factors that might potentially contribute to overprescribing, such as microcytic anemia and hiatal hernias.

### Standard Protocol Approvals, Registrations, and Participant Consent

The study was conducted in accordance with the ethical standards set forth in the Helsinki Declaration (1983). The local ethical committee of Nantes (France) approved the study protocol. The protocol was compliant with the Strengthening the Reporting of Observational Studies in Epidemiology statement guidelines. The waive of consent for this study was authorized in accordance with the French law. All participants provided written informed consent.

### Statistics

The subjects’ baseline characteristics were summarized using the means and standard deviations or frequencies and percentages, as appropriate. The normality of the data distribution was verified using the skewness-kurtosis test. Because the number of observations was >40 for each group, no transformations were applied to the variables of interest. Subjects were separated into two groups based on the overuse or no overuse of a PPI. Between-group comparisons were performed using an independent samples *t*-test or Chi-square test, as appropriate. Univariate and multiple linear logistic analyses were performed to specify the association between overuse (dependent variable) and multimorbidities (independent variable), adjusted by the subjects' baseline characteristics. Odds ratios (OR) with 95% confidence intervals (95% CI) were presented. P-values less than 0.05 were considered to be statistically significant. All statistics were performed using the SPSS software (version 17.0; SPSS, Inc., Chicago, IL, USA).

## Results

Two hundred and eighty individuals aged 75 and older (mean age 86.4 ± 7.4 years; 70% female) being treated with a PPI were included. Overprescribing of a PPI was found in 207 (73.9%) patients. The mean age of those overprescribed a PPI was 86.7 ± 7.9 years; 147 patients (71%) were women, and 48 patients (23.2%) lived in a nursing home. The number of concomitant medications was higher in patients who were overprescribed a PPI than in patients with an appropriately prescribed PPI (p = 0.018). An overprescription of PPIs was associated with cardiac diseases (p < 0.001), metabolic diseases (p = 0.048), esogastric diseases (p = 0.004), vascular diseases (p = 0.029), a burden of multimorbidities with CIRS (p = 0.031), and with cortisteroids’ treatment (p = 0.002; Table 1).

**Table 1:** Characteristics of patients according to overprescription of a PPI (n = 280).

Characteristics	Overprescription of PPI		p-value*
	No(n = 73)	Yes(n = 207)	
Age (years), mean ± SD	85.4 ± 5.4	86.7 ± 7.9	0.173
Female sex, n (%)	47 (64.4)	147 (71)	0.304
Living in nursing home, n (%)	12 (16.4)	48 (23.2)	0.250
BMI, mean ± SD	25.8 ± 4.7	25.4 ±5.1	0.706
ADL/6, mean ± SD	4.12 ± 1.9	3.9 ±1.9	0.385
Number of concomitant medications, mean ±SD	8.1 ± 2.7	9.0 ± 2.8	**0.018**
Full dose of PPI, n (%)	35 (47.9)	74 (35.7)	0.071
Microcytic anemia, n (%)	2 (2.7)	17 (8.2)	0.174
Hiatal hernias, n (%)	10 (13.7)	33 (15.9)	0.709
Type of concomitant medications			
Antiaggregants, n (%)	33 (45.2)	99 (47.8)	0.685
Cortisteroids’, n (%)	2 (2.7)	34 (16.5)	**0.002**
Anticoagulants, n (%)	19 (26)	55 (26.6)	0.911
Associated multimorbidities			
CIRS, mean ± SD	9.6 ± 3.4	10.8 ± 3.6	**0.031**
Cardiac diseases, n (%)	58 (79.5)	197 (95.2)	**<0.001**
Metabolic diseases, n (%)	19 (26)	81 (39.1)	**0.048**
Esogastric diseases, n (%)	42 (57.5)	78 (37.7)	**0.004**
Vascular diseases, n (%)	30 (41.1)	117 (56.5)	**0.029**

*based on independent samples Chi-square or *t-*test as appropriate, with *P* significant at ≤0.05; significant *P*-values (i.e. ≤0.05) are indicated in bold

An unadjusted model showed a statistically significant association between the overprescription of a PPI and the number of concomitant medications (OR = 1.13, 95% CI = 1.02–1.25 p = 0.019), treatment by corticosteroids’ (OR = 7.02, 95% CI = 1.64–29.99, p = 0,009), cardiac diseases (OR = 5.09, 95% CI = 2.17–11.94, p < 0.001), metabolic diseases (OR = 1.83, 95% CI = 1.01–3.30, p = 0.046), vascular diseases (OR = 1.86, 95% CI = 1.08–3.2, p = 0.024), and the burden of multimorbidities with CIRS (OR = 1.08, 95% CI = 1.01–1.17, p = 0,032) but not with antiaggregants (p = 0.675) or anticoagulants (p = 0.911).

The over prescription of a PPI and the esogastric diseases (OR = 0.45, 95% CI = 0.26–0.77, p = 0.004) were negatively associated (Table 2).

**Table 2:** Uni- and multivariate logistic regression models showing the association between overprescription of a PPI and individual multimorbidities adjusted for clinical characteristics (n = 280).

Characteristics	Unadjusted model			Fully adjusted model		
	OR	CI 95%	p-value*	OR	CI 95%	p-value*
Age	1.024	[0.99–1.06]	0.197	1.020	[0.98–1.06]	0.318
Female sex	1.008	[0.41–1.30]	0.292	1.365	[0.70–2.67]	0.364
Living in nursing home	1.535	[0.76–3.08]	0.229	1.308	[0.58–2.93]	0.515
BMI	0.985	[0.91–1.06]	0.704	-	-	-
ADL/6	0.938	[0.81–1.08]	0.384	-	-	-
Number of concomitant medications	1.128	[1.02–1.25]	**0.019**	1.086	[0.96–1.22]	0.184
Full dose of PPI	0.604	[0.35–1.04]	0.067	0.647	[0.34–1.22]	0.176
Microcytic anemia	3.210	[0.72–14.25]	0.125	3.823	[0.72–20.37]	0.116
Hiatal hernias	1.202	[0.56–2.58]	0.637	-	-	-
Type of concomitant medications						
Antiaggregants,	1.121	[0.66–1.92]	0.675	0.686	[0.31–1.53]	0.357
Corticosteroids,	7.017	[1.64–29.99]	**0.009**	5.390	[1.20–24.27]	**0.028**
Anticoagulants,	1.035	[0.56–1.90]	0.911	0.735	[0.31–1.67]	0.490
Associated multimorbidities						
CIRS	1.084	[1.01–1.17]	**0.032**	0.994	[0.88–1.12]	0.923
Cardiac diseases	5.095	[2.17–11.94]	**<0.001**	4.171	[1.41–12.38]	**0.010**
Metabolic diseases	1.830	[1.01–3.30]	**0.046**	2.143	[1.03–4.47]	**0.042**
Esogastric diseases	0.446	[0.26–0.77]	**0.004**	0.491	[0.26–0.94]	**0.033**
Vascular diseases	1.860	[1.08–3.20]	**0.024**	1.890	[0.93–3.83]	0.077

*based on independent samples chi-square or *t-*test as appropriate, with *P* significant at ≤0.05; significant *P*-values (i.e., ≤0.05) are indicated in bold

In the fully adjusted model, cardiac diseases (OR = 4.17, 95% CI = 1.41–12.38, p = 0.010), metabolic diseases (OR = 2.14, 95% CI = 1.03–4.47, p = 0.042), and treatment by corticosteroids’ (OR = 5.39, 95% CI = 1.20–24.27, p = 0.028) were statistically associated with an overprescription of a PPI. Esogastric diseases (OR = 0.49, 95% CI = 0.26–0.94, p = = 0.033) were negatively associated with an overprescription of a PPI (Table 2).

## Discussion

This study found an association between cardiac diseases, metabolic diseases, and esogastric diseases and the overprescription of a PPI in patients aged 75 and older. Cortisteroids’ treatments were also associated with overprescribing.

In our study, we showed that cardiac diseases were significantly associated with the overprescription of a PPI. To our knowledge, this association has not been previously documented in the literature. One explanation for this result may be that the patients who have a history of congestive heart failure, ischemic heart disease, or unspecified chest pain might have been hospitalized in an intensive care unit (ICU) in previous years [17]. Patients admitted to the ICU are commonly prescribed a PPI for stress ulcer prophylaxis, contributing to an inappropriate and controllable overutilization [18]. This is supported by several trials that have demonstrated significant overutilization of PPIs outside of the ICU [19, 20].

In the current study, the association noted between metabolic disease and the overprescription of a PPI was surprising. This association has not been previously documented in the literature, but this link might have several different explanations. In the literature, the prevalence of gastrointestinal symptoms is reported to be higher in patients with diabetes mellitus than the general population [21, 22]. Moreover, patients with diabetes mellitus take oral hypoglycemic agents, such as metformin. Metformin is a commonly used antidiabetic drug. However, this treatment has common side effects, such as gastrointestinal symptoms (abdominal pain, nausea, vomiting, diarrhea, and bloating) affecting up to 30% of patients in some studies [23]. Therefore, a PPI may be prescribed to reduce the side effects caused by metformin.

In the current study, esogastric diseases were negatively associated with a risk of overprescribing a PPI. This is consistent with studies that show that PPIs are first choice in the treatment of esogastric diseases, such as GERD peptic ulcers, erosive esophagitis, and dyspepsia, because PPIs are the most potent medications currently available for the reduction of gastric acid secretion [24, 25].

In the current study, we found that in the multivariate analysis, the burden of multimorbidities was not significantly associated with an overprescription of a PPI, unlike in the univariate analysis; however, in the literature, studies exploring the burden of multimorbidities using the CCI, CIRS, or the number of multimorbidities are inconsistent [5, 6, 12, 26]. These results could be explained by the use of different methodologies that employ various scales; however, in previous studies, each individual multimorbidity included in the scale was not evaluated in the multivariate model, contrary to our study.

In our study, corticosteroids’ were significantly associated with the overprescription of a PPI. This conclusion is in agreement with the results of studies, which found that the most common overprescription for the acid suppressive therapy was peptic ulcer prophylaxis during corticoid steroid therapy [27,28]. The use of corticoids is commonly associated with the use of PPI prophylaxis to prevent gastrointestinal bleeding; however, no scientific evidence exists suggesting the need for this therapy in the absence of other risks factors.

In our study, we did not find an association with antiaggregants, while the results regarding antiaggregants in the previous studies have been mixed. A French study showed that the antiaggregant (43.1%) was the principal cause for the overuse of a PPI [12]. By contrast, other studies have not found a significant association with antiaggregants and the overprescription of a PPI [6, 25].

In the current study, anticoagulants were not associated with an overprescription of a PPI. This association in the literature is conflicting. A retrospective study with 108 patients showed that concomitant anticoagulant medications were associated with an acid suppressive therapy [26], however, other studies have demonstrated that anticoagulation was a factor that significantly decreased the risk of PPI overprescribing [6, 25].

The current study was a multicenter, prospective study; nevertheless, some limitations should be considered. First, the assessment of the appropriateness of the drug recommendation is solely based on the information available from the medical data provided by the general practitioner; consequently, the numbers for an overprescription of a PPI may be overestimated. Second, occasionally, the indication was uncertain because of a history of gastritis or dyspepsia, which might overestimate the incidences of an overprescription of a PPI. Third, the small number of subjects studied might have limited the reliability or precision of results. Finally, there is no international consensus for the appropriate prescription of a PPI. This study was based on the French recommendations by AFFSAPS. However, the high rate of overprescription of PPIs (73, 9%) observed in our study is consistent with the rates (between 61% and 85%) published by others [5, 6].

In conclusion, this study showed an association between cardiac diseases, metabolic diseases, and the overprescription of PPI in patients aged 75 and older. Additional prospective studies are required to confirm these results.

## Supporting Information

S1 Dataset multimorbidities and overprescription of proton pump inhibitors in older patients. (PDF)
