# Supplementary material for: Multimorbidities and Overprescription of Proton Pump Inhibitors in Older Patients
Source: PLoS One. 2015 Nov 4;10(11):e0141779. doi: 10.1371/journal.pone.0141779 (PMC4633104; doi:10.1371/journal.pone.0141779)
Supplement: S1 Dataset — (PDF) [file pone.0141779.s001.pdf]

N	Sexe	Age	MotifCSG	CSG_1	CSG_2	CSG_3	CSG_4	CSG_5
1	1	83	2	0	1	0	0	0
2	2	94	1	1	0	0	0	0
3	2	78	9	0	0	0	0	0
4	2	90	9	0	0	0	0	0
5	2	89	1	1	0	0	0	0
6	2	87	5	0	0	0	0	1
7	2	84	8	0	0	0	0	0
8	2	103	2	0	1	0	0	0
9	2	95	3	0	0	1	0	0
10	2	88	5	0	0	0	0	1
11	1	88	5	0	0	0	0	1
12	1	87	1	1	0	0	0	0
13	2	82	3	0	0	1	0	0
14	2	101	5	0	0	0	0	1
15	1	82	8	0	0	0	0	0
16	2	89	2	0	1	0	0	0
17	2	89	1	1	0	0	0	0
18	2	94	2	0	1	0	0	0
19	2	80	9	0	0	0	0	0
20	1	96	2	0	1	0	0	0
21	1	79	6	0	0	0	0	0
22	1	89	9	0	0	0	0	0
23	2	95	7	0	0	0	0	0
24	2	82	2	0	1	0	0	0
25	2	93	3	0	0	1	0	0
26	1	81	2	0	1	0	0	0
27	2	91	1	1	0	0	0	0
28	1	90	7	0	0	0	0	0
29	2	100	2	0	1	0	0	0
30	1	78	2	0	1	0	0	0
31	2	89	2	0	1	0	0	0
32	2	87	9	0	0	0	0	0
33	2	88	3	0	0	1	0	0
34	2	88	1	1	0	0	0	0
35	1	91	2	0	1	0	0	0
36	2	9	8	0	0	0	0	0
37	2	92	2	0	1	0	0	0
38	2	90	7	0	0	0	0	0
39	1	80	7	0	0	0	0	0
40	2	80	6	0	0	0	0	0
41	1	86	1	1	0	0	0	0
42	2	86	1	1	0	0	0	0
43	2	77	8	0	0	0	0	0
44	2	78	1	1	0	0	0	0
45	2	92	2	0	1	0	0	0
46	2	97	1	1	0	0	0	0
47	2	85	1	1	0	0	0	0
48	2	94	2	0	1	0	0	0
49	1	91	2	0	1	0	0	0
50	2	94	2	0	1	0	0	0
51	2	84	8	0	0	0	0	0
52	2	82	8	0	0	0	0	0
53	2	85	8	0	0	0	0	0

54	2	79	5	0	0	0	0	1
55	2	92	6	0	0	0	0	0
56	2	88	8	0	0	0	0	0
57	2	75	4	0	0	0	1	0
58	2	90	6	0	0	0	0	0
59	2	96	2	0	1	0	0	0
60	2	92	3	0	0	1	0	0
61	2	86	2	0	1	0	0	0
62	2	79	7	0	0	0	0	0
63	2	80	7	0	0	0	0	0
64	2	83	8	0	0	0	0	0
65	2	92	8	0	0	0	0	0
66	1	81	2	0	1	0	0	0
67	2	85	3	0	0	1	0	0
68	2	87	2	0	1	0	0	0
69	2	85	6	0	0	0	0	0
70	2	83	8	0	0	0	0	0
71	2	96	6	0	0	0	0	0
72	2	90	5	0	0	0	0	1
73	2	87	8	0	0	0	0	0
74	2	90	8	0	0	0	0	0
6	1	91	1	1	0	0	0	0
76	2	87	2	0	1	0	0	0
77	2	87	5	0	0	0	0	1
78	1	79	6	0	0	0	0	0
79	1	83	1	1	0	0	0	0
80	1	91	9	0	0	0	0	0
81	2	87	4	0	0	0	1	0
82	2	88	8	0	0	0	0	0
83	2	87	1	1	0	0	0	0
84	2	92	5	0	0	0	0	1
85	2	92	3	0	0	1	0	0
87	1	87	1	1	0	0	0	0
88	2	86	9	0	0	0	0	0
89	2	75	2	0	1	0	0	0
90	2	93	3	0	0	1	0	0
91	2	91	7	0	0	0	0	0
92	2	86	6	0	0	0	0	0
93	2	91	1	1	0	0	0	0
94	2	89	3	0	0	1	0	0
95	1	92	9	0	0	0	0	0
96	1	89	3	0	0	1	0	0
97	2	87	5	0	0	0	0	1
98	1	105	9	0	0	0	0	0
99	2	91	9	0	0	0	0	0
100	2	88	5	0	0	0	0	1
101	1	92	8	0	0	0	0	0
102	1	80	1	1	0	0	0	0
103	2	88	2	0	1	0	0	0
104	2	85	7	0	0	0	0	0
105	2	87	8	0	0	0	0	0
106	2	86	5	0	0	0	0	1
107	1	87	5	0	0	0	0	1
108	2	86	2	0	1	0	0	0
109	1	93	7	0	0	0	0	0
110	2	92	3	0	0	1	0	0

111	2	97	1	1	0	0	0	0
112	1	88	2	0	1	0	0	0
113	1	90	9	0	0	0	0	0
114	1	76	8	0	0	0	0	0
115	1	93	2	0	1	0	0	0
116	2	82	1	1	0	0	0	0
117	2	92	8	0	0	0	0	0
118	1	79	8	0	0	0	0	0
119	2	92	8	0	0	0	0	0
120	2	79	9	0	0	0	0	0
121	2	88	9	0	0	0	0	0
122	2	87	4	0	0	0	1	0
123	1	91	8	0	0	0	0	0
124	1	82	1	1	0	0	0	0
125	2	93	3	0	0	1	0	0
126	1	81	2	0	1	0	0	0
127	1	80	9	0	0	0	0	0
128	2	80	1	1	0	0	0	0
129	1	94	2	0	1	0	0	0
130	2	83	3	0	0	1	0	0
131	2	85	1	1	0	0	0	0
132	2	78	8	0	0	0	0	0
133	1	86	6	0	0	0	0	0
134	2	80	8	0	0	0	0	0
135	2	79	3	0	0	1	0	0
136	2	83	9	0	0	0	0	0
137	2	95	1	1	0	0	0	0
138	2	90	8	0	0	0	0	0
139	2	88	9	0	0	0	0	0
140	2	90	8	0	0	0	0	0
141	2	83	8	0	0	0	0	0
142	2	87	2	0	1	0	0	0
143	2	93	1	1	0	0	0	0
144	2	84	3	0	0	1	0	0
145	2	92	8	0	0	0	0	0
146	1	92	2	0	1	0	0	0
147	1	87	8	0	0	0	0	0
148	1	76	6	0	0	0	0	0
149	1	74	1	1	0	0	0	0
150	2	77	7	0	0	0	0	0
151	1	73	7	0	0	0	0	0
152	2	81	7	0	0	0	0	0
153	2	85	1	1	0	0	0	0
154	2	88	7	0	0	0	0	0
155	2	92	8	0	0	0	0	0
156	1	94	7	0	0	0	0	0
157	1	81	6	0	0	0	0	0
158	1	85	8	0	0	0	0	0
159	2	88	9	0	0	0	0	0
160	1	87	4	0	0	0	1	0
161	2	88	7	0	0	0	0	0
162	1	85	7	0	0	0	0	0
163	2	101	1	1	0	0	0	0
164	1	81	7	0	0	0	0	0
165	2	85	5	0	0	0	0	1
166	1	78	8	0	0	0	0	0

167	2	84	7	0	0	0	0	0
168	2	90	1	1	0	0	0	0
169	2	81	1	1	0	0	0	0
170	2	82	1	1	0	0	0	0
171	2	87	6	0	0	0	0	0
172	2	78	7	0	0	0	0	0
173	2	83	7	0	0	0	0	0
174	2	99	1	1	0	0	0	0
175	2	92	8	0	0	0	0	0
176	2	83	8	0	0	0	0	0
177	2	90	8	0	0	0	0	0
178	2	96	1	1	0	0	0	0
179	2	88	8	0	0	0	0	0
180	1	77	8	0	0	0	0	0
181	2	89	1	1	0	0	0	0
182	2	89	8	0	0	0	0	0
183	1	93	1	1	0	0	0	0
184	2	90	5	0	0	0	0	1
185	2	92	1	1	0	0	0	0
187	2	77	7	0	0	0	0	0
188	2	82	6	0	0	0	0	0
189	2	83	3	0	0	1	0	0
190	2	89						
191	2	86	6	0	0	0	0	0
192	2	86	9	0	0	0	0	0
193	1	85	7	0	0	0	0	0
194	2	92	1	1	0	0	0	0
195	1	92	8	0	0	0	0	0
196	2	87	1	1	0	0	0	0
197	2	97	4	0	0	0	1	0
198	2	84	8	0	0	0	0	0
199	1	88	1	1	0	0	0	0
200	2	88	8	0	0	0	0	0
201	1	87	4	0	0	0	1	0
202	1	78	2	0	1	0	0	0
203	2	88	2	0	1	0	0	0
204	2	80	7	0	0	0	0	0
205	2	88	3	0	0	1	0	0
206	1	90	5	0	0	0	0	1
207	2	79	5	0	0	0	0	1
208	2	90	2	0	1	0	0	0
209	2	81	9	0	0	0	0	0
210	1	83	7	0	0	0	0	0
211	2	88	2	0	1	0	0	0
212	1	88	4	0	0	0	1	0
213	2	81	6	0	0	0	0	0
214	2	88	3	0	0	1	0	0
215	2	83	3	0	0	1	0	0
216	1	92	8	0	0	0	0	0
217	2	82	7	0	0	0	0	0
218	2	84	8	0	0	0	0	0
219	2	85	7	0	0	0	0	0
220	1	83	1	1	0	0	0	0
221	1	84	6	0	0	0	0	0
222	2	96	9	0	0	0	0	0
223	2	91	8	0	0	0	0	0

224	2	91	3	0	0	1	0	0
225	1	93	3	0	0	1	0	0
226	2	85	8	0	0	0	0	0
227	2	76	8	0	0	0	0	0
228	1	83	1	1	0	0	0	0
229	2	94	9	0	0	0	0	0
230	2	79	7	0	0	0	0	0
231	2	83	8	0	0	0	0	0
232	2	77	2	0	1	0	0	0
233	1	93	9	0	0	0	0	0
234	2	78	9	0	0	0	0	0
235	1	81	6	0	0	0	0	0
236	2	82	8	0	0	0	0	0
237	2	87	6	0	0	0	0	0
238	1	81	3	0	0	1	0	0
239	1	82	2	0	1	0	0	0
240	2	87	8	0	0	0	0	0
241	2	98	8	0	0	0	0	0
242	2	82	8	0	0	0	0	0
244	1	82	6	0	0	0	0	0
245	1	87	7	0	0	0	0	0
246	1	81	7	0	0	0	0	0
247	2	88	8	0	0	0	0	0
248	2	93	1	1	0	0	0	0
249	2	95	8	0	0	0	0	0
250	2	88	9	0	0	0	0	0
251	2	88	8	0	0	0	0	0
252	2	93	1	1	0	0	0	0
254	1	86	7	0	0	0	0	0
255	2	82	2	0	1	0	0	0
256	1	87	1	1	0	0	0	0
257	1	89	9	0	0	0	0	0
258	1	85	7	0	0	0	0	0
259	1	85	3	0	0	1	0	0
262	1	82	7	0	0	0	0	0
263	2	98	9	0	0	0	0	0
264	1	78	7	0	0	0	0	0
265	2	85	5	0	0	0	0	1
266	2	80	8	0	0	0	0	0
267	2	91	7	0	0	0	0	0
268	2	92	8	0	0	0	0	0
269	2	86	1	1	0	0	0	0
270	1	82	9	0	0	0	0	0
271	2	85	6	0	0	0	0	0
272	1	90	6	0	0	0	0	0
273	1	89	9	0	0	0	0	0
274	1	86	7	0	0	0	0	0
275	1	76	9	0	0	0	0	0
276	2	90	6	0	0	0	0	0
277	2	79	6	0	0	0	0	0
278	2	92	2	0	1	0	0	0
279	2	78	1	1	0	0	0	0
280	2	76	9	0	0	0	0	0
281	1	88	1	1	0	0	0	0
282	2	85	6	0	0	0	0	0
283	1	92	7	0	0	0	0	0

284	2	83	2	0	1	0	0	0
285	2	87	7	0	0	0	0	0
286	2	82	8	0	0	0	0	0

CSG_6	CSG_7	CSG_8	CSG_9	CSG_10	ADL	Poids	Taille
0	0	0	0	0	5,0	69,0	170,0
0	0	0	0	0	4,0	56,0	157,0
0	0	0	1	0	6,0	69,0	148,0
0	0	0	1	0	1,5	65,0	138,0
0	0	0	0	0	0,5	74,0	160,0
0	0	0	0	0	5,5	49,0	144,0
0	0	1	0	0	6,0	45,0	149,0
0	0	0	0	0	1,5	51,0	143,0
0	0	0	0	0	6,0	55,0	150,0
0	0	0	0	0	1,5	56,0	
0	0	0	0	0	0,0	59,0	162,0
0	0	0	0	0	6,0	58,0	162,0
0	0	0	0	0	4,5	66,0	156,0
0	0	0	0	0	4,5	40,0	140,0
0	0	1	0	0	5,5	89,0	170,0
0	0	0	0	0	6,0	63,0	149,0
0	0	0	0	0	0,5		
0	0	0	0	0	3,0	44,0	141,0
0	0	0	1	0	3,5	68,0	153,0
0	0	0	0	0	4,5	53,0	160,0
1	0	0	0	0	3,0	91,0	164,0
0	0	0	1	0	0,5	67,0	181,0
0	1	0	0	0	2,0	46,0	165,0
0	0	0	0	0	5,0	93,0	
0	0	0	0	0	5,0	81,0	
0	0	0	0	0	5,0	125,0	
0	0	0	0	0	6,0	45,0	147,0
0	1	0	0	0	4,0	58,0	164,0
0	0	0	0	0	3,0	56,9	
0	0	0	0	0	2,0	66,0	174,0
0	0	0	0	0	4,0	65,0	149,0
0	0	0	1	0	2,5	49,5	146,0
0	0	0	0	0	5,0	69,0	151,0
0	0	0	0	0	0,5		
0	0	0	0	0	5,5	84,0	172,0
0	0	1	0	0	5,0	59,0	149,0
0	0	0	0	0	5,0	74,5	151,0
0	1	0	0	0	0,0	72,0	147,0
0	1	0	0	0	3,5	78,0	180,0
1	0	0	0	0	0,0	50,0	
0	0	0	0	0	6,0	77,0	
0	0	0	0	0	5,0	69,0	
0	0	1	0	0	2,5	60,0	
0	0	0	0	0	3,5	42,0	160,0
0	0	0	0	0	2,5	67,8	
0	0	0	0	0	3,0	70,0	137,0
0	0	0	0	0	3,0	59,0	145,0
0	0	0	0	0	5,5	72,4	146,0
0	0	0	0	0	2,0	55,0	168,0
0	0	0	0	0	6,0	63,2	155,0
0	0	1	0	0	0,5	70,0	
0	0	1	0	0	6,0	54,1	
0	0	1	0	0	6,0	61,2	152,0

0	0	0	0	0	6,0	59,0	160,0
1	0	0	0	0	3,5	51,0	
0	0	1	0	0	2,0	62,0	
0	0	0	0	0	6,0	104,0	
1	0	0	0	0	3,5	57,7	
0	0	0	0	0	0,5	50,0	
0	0	0	0	0	5,5	61,3	150,0
0	0	0	0	0	5,5	73,0	149,0
0	1	0	0	0	3,5	42,2	148,0
0	1	0	0	0	4,5	68,0	
0	0	1	0	0	6,0	67,0	158,0
0	0	1	0	0	2,5	97,5	
0	0	0	0	0	3,0	72,7	178,0
0	0	0	0	0	6,0	50,0	
0	0	0	0	0	3,0	60,0	
1	0	0	0	0	1,5	53,0	
0	0	1	0	0	5,5	59,0	152,0
1	0	0	0	0	3,0	66,6	139,0
0	0	0	0	0	5,0	46,0	153,0
0	0	1	0	0	4,0		
0	0	1	0	0	3,0	50,0	160,0
0	0	0	0	0	3,5	66,0	
0	0	0	0	0	1,0	57,0	
0	0	0	0	0	6,0	57,0	
1	0	0	0	0	3,5	77,9	164,0
0	0	0	0	0	0,5	64,0	
0	0	0	1	0	5,0	67,0	132,0
0	0	0	0	0	3,0	43,0	
0	0	1	0	0	4,0		
0	0	0	0	0	6,0		
0	0	0	0	0	3,0	72,5	
0	0	0	0	0	4,5	45,0	
0	0	0	0	0	3,0		
0	0	0	1	0	4,0	32,0	
0	0	0	0	0	5,5	102,0	
0	0	0	0	0	6,0		
0	1	0	0	0	2,0	61,2	138,0
1	0	0	0	0	2,0	44,3	158,0
0	0	0	0	0	2,5	47,2	151,0
0	0	0	0	0	5,0	47,3	
0	0	0	1	0	3,5		
0	0	0	0	0	1,5	71,8	
0	0	0	0	0	1,5	53,5	
0	0	0	1	0	1,0	52,0	
0	0	0	1	0	3,0	62,0	
0	0	0	0	0	6,0		
0	0	1	0	0		59,4	
0	0	0	0	0	2,0	98,0	174,0
0	0	0	0	0	4,0	65,9	151,0
0	1	0	0	0	2,0	34,8	149,0
0	0	1	0	0	5,5	67,6	
0	0	0	0	0	6,0	46,6	
0	0	0	0	0	0,0	49,0	156,0
0	0	0	0	0	2,5	56,0	145,0
0	1	0	0	0	3,0	120,0	176,0
0	0	0	0	0	1,5	60,9	158,0

0	0	0	0	0	4,0	66,0	155,0
0	0	0	0	0		58,8	
0	0	0	1	0	4,0	50,9	
0	0	1	0	0	3,5	85,0	170,0
0	0	0	0	0	1,0		
0	0	0	0	0	5,0	73,1	
0	0	1	0	0	3,5	91,5	
0	0	1	0	0	3,0	81,0	
0	0	1	0	0	5,5	59,0	
0	0	0	1	0	5,5	91,0	
0	0	0	1	0	4,0	60,0	
0	0	0	0	0	6,0	76,3	156,0
0	0	1	0	0	3,5	72,3	158,0
0	0	0	0	0	1,0	66,0	
0	0	0	0	0	5,5	47,8	
0	0	0	0	0	5,5	71,0	
0	0	0	1	0	5,0	60,0	
0	0	0	0	0	2,0	57,6	
0	0	0	0	0	1,5	63,0	
0	0	0	0	0	5,5	56,0	155,0
0	0	0	0	0	6,0	48,0	
0	0	1	0	0	5,5	49,0	
1	0	0	0	0	4,5		
0	0	1	0	0	6,0	65,3	
0	0	0	0	0	4,5	39,3	
0	0	0	1	0	4,0		
0	0	0	0	0	6,0	43,5	
0	0	1	0	0	1,0		
0	0	0	1	0	6,0	49,6	
0	0	1	0	0	1,0		
0	0	1	0	0	5,5	65,5	
0	0	0	0	0	5,5	70,0	155,0
0	0	0	0	0	5,5	68,5	140,0
0	0	0	0	0	5,0	73,4	
0	0	1	0	0	1,0		
0	0	0	0	0	0,5		
0	0	1	0	0	4,0	81,0	172,0
1	0	0	0	0	6,0	52,0	150,0
0	0	0	0	0	4,0	72,0	
0	1	0	0	0	6,0	67,0	155,0
0	1	0	0	0	5,0	84,1	
0	1	0	0	0	6,0		
0	0	0	0	0	4,0	50,0	148,0
0	1	0	0	0	0,0	46,0	
0	0	1	0	0	3,0	63,0	
0	1	0	0	0	3,6	60,0	
1	0	0	0	0	6,0	63,0	171,0
0	0	1	0	0	4,5	66,0	
0	0	0	1	0	6,0		
0	0	0	0	0	1,0	81,0	
0	1	0	0	0	5,5	56,0	160,0
0	1	0	0	0	3,0	97,0	172,0
0	0	0	0	0	3,0	56,0	150,0
0	1	0	0	0	5,0	75,0	152,0
0	0	0	0	0	5,0		
0	0	1	0	0	4,5	75,0	

0	1	0	0	0	0,0	53,0	
0	0	0	0	0	6,0		
0	0	0	0	0	1,0	54,0	
0	0	0	0	0	6,0		
1	0	0	0	0	6,0	41,0	
0	1	0	0	0	2,5		
0	1	0	0	0	6,0	60,0	
0	0	0	0	0	3,5	44,0	
0	0	1	0	0	6,0		
0	0	1	0	0	4,5	44,0	
0	0	1	0	0	0,5		
0	0	0	0	0	6,0	51,0	150,0
0	0	1	0	0	4,5	48,0	
0	0	1	0	0	5,0	58,0	160,0
0	0	0	0	0	0,0	80,0	
0	0	1	0	0	2,0	57,0	160,0
0	0	0	0	0	5,5	66,8	164,0
0	0	0	0	0	6,0		
0	0	0	0	0	4,0	67,7	
0	1	0	0	0	6,0		
1	0	0	0	0	5,5	59,5	165,0
0	0	0	0	0	6,0	67,0	152,0
					0,5		
1	0	0	0	0	6,0	42,0	
0	0	0	1	0	6,0		
0	1	0	0	0	4,5	67,0	
0	0	0	0	0	0,0	50,5	
0	0	1	0	0	5,5	61,4	
0	0	0	0	0	1,0	60,3	
0	0	0	0	0	3,5		
0	0	1	0	0	6,0	43,0	
0	0	0	0	0	0,0	57,5	
0	0	1	0	0	5,5	43,4	
0	0	0	0	0	0,5	81,0	
0	0	0	0	0	2,0		
0	0	0	0	0	2,5	47,0	
0	1	0	0	0	6,0	63,3	
0	0	0	0	0	5,5	74,0	160,0
0	0	0	0	0	4,5	60,0	
0	0	0	0	0	6,0		
0	0	0	0	0	2,0		
0	0	0	1	0	6,0	56,0	163,0
0	1	0	0	0	5,5	54,8	160,0
0	0	0	0	0	2,0	54,0	155,0
0	0	0	0	0	3,5	58,0	169,0
1	0	0	0	0	5,0	56,0	158,0
0	0	0	0	0	5,5	43,0	146,0
0	0	0	0	0	6,0	53,0	151,0
0	0	1	0	0	3,5	65,0	168,0
0	1	0	0	0	5,5	42,0	162,0
0	0	1	0	0	2,5	65,0	150,0
0	1	0	0	0	6,0	59,0	168,0
0	0	0	0	0	4,5	95,3	170,0
1	0	0	0	0	1,5	72,1	178,0
0	0	0	1	0	6,0	54,3	
0	0	1	0	0	3,5	60,2	158,0

0	0	0	0	0	6,0	45,7	
0	0	0	0	0	3,0	55,0	175,0
0	0	1	0	0	6,0	64,2	154,0
0	0	1	0	0	6,0	58,7	155,0
0	0	0	0	0	5,0	63,0	162,0
0	0	0	1	0	2,5	73,0	158,0
0	1	0	0	0	1,5	87,9	155,0
0	0	1	0	0	4,5	71,6	
0	0	0	0	0	6,0	66,3	165,0
0	0	0	1	0	5,0	59,6	142,0
0	0	0	1	0	6,0		156,0
1	0	0	0	0	4,0	72,5	
0	0	1	0	0	3,0	49,4	
1	0	0	0	0	6,0	42,9	
0	0	0	0	0	6,0	86,0	175,0
0	0	0	0	0	1,0	89,5	180,0
0	0	1	0	0	4,5	74,0	155,0
0	0	1	0	0	5,0	52,8	146,0
0	0	1	0	0	6,0	57,4	155,0
1	0	0	0	0	6,0	56,5	153,0
0	1	0	0	0	3,0	63,5	
0	1	0	0	0	5,0		
0	0	1	0	0	2,5	103,0	155,0
0	0	0	0	0	0,5	76,0	
0	0	1	0	0	5,5	68,7	163,0
0	0	0	1	0	6,0	54,2	160,0
0	0	1	0	0	1,5	52,8	153,0
0	0	0	0	0	0,5	46,1	
0	1	0	0	0	4,0	62,0	163,0
0	0	0	0	0	6,0	45,0	157,0
0	0	0	0	0	5,5	70,0	156,0
0	0	0	1	0	0,0	55,0	
0	1	0	0	0	2,0	76,6	172,0
0	0	0	0	0	6,0	72,6	160,0
0	1	0	0	0	2,0	103,0	181,0
0	0	0	1	0	5,5	78,7	
0	1	0	0	0	4,5	80,0	160,0
0	0	0	0	0	5,0	62,0	170,0
0	0	1	0	0	3,5	58,0	150,0
0	1	0	0	0	6,0	71,0	155,0
0	0	1	0	0	6,0	50,0	156,0
0	0	0	0	0	6,0	60,9	16,0
0	0	0	1	0	6,0	70,0	170,0
1	0	0	0	0	2,0	96,0	158,0
1	0	0	0	0	2,5	74,0	
0	0	0	1	0	6,0	80,0	180,0
0	1	0	0	0	6,0	86,0	178,0
0	0	0	1	0	6,0	80,0	165,0
1	0	0	0	0	1,0	59,8	165,0
1	0	0	0	0	5,5	77,0	157,0
0	0	0	0	0	2,5	62,7	165,0
0	0	0	0	0	6,0	49,0	150,0
0	0	0	1	0	3,0	69,0	157,0
0	0	0	0	0	2,5	52,0	166,0
1	0	0	0	0	5,5	76,0	162,0
0	1	0	0	0	5,5	60,8	

0	0	0	0	0	6,0	60,0	157,0
0	1	0	0	0	5,5	64,2	169,0
0	0	1	0	0	6,0	39,2	150,0

BMI	Hauteur T-G (cm)	LieuHab	DomSeul	AideDom	TypAideDom
23,9	54,0	1	1	1	1
22,8	48,0	2		1	
20,5	45,0	1	1	1	2
34,0	41,0	2		1	
29,0	53,0	2		1	
23,8	44,0	1	1	1	1
20,3	46,0	1	1	1	1
25,0	45,0	1	1	1	1
24,0		1	0	0	
		2		1	
22,0	50,0	2		1	
18,7	52,0	1	1	1	3
27,0	47,0	1	0	1	1
20,0	43,0	1	0	1	2
31,0	55,0	1	1	1	1
28,0	47,0	1	1	1	1
		2		1	
22,0	43,0	2		1	
29,0	46,0	1	0	0	
20,0		2			
34,0	52,0	1	0	0	
20,8		2		1	
17,0		1	0	1	2
		1	1	1	1
		1	1	1	1
		1	0	1	1
21,0		1	1	1	1
21,0	51,0	1	0	1	1
		1	1	1	1
22,0	56,0	1	0	1	1
29,0	47,0	1	1	1	1
23,0	44,0	1	1	1	2
30,0	48,0	2		1	
	44,0	2		1	
28,0	55,0	1	1	0	
26,6	47,0	1	1	1	3
32,0	45,0	2		1	
33,0	46,0	2		1	
24,0		1	1	1	1
		2		1	
		1	0	1	1
		1	1	1	2
		1	1	1	1
16,7	51,0	1	1	1	3
		2		1	
37,0	41,0	2		1	
28,0	44,0	1	0	1	3
34,0	46,0	1	1	1	3
19,5	53,0	1	0	1	3
26,0	51,0	1	0	1	2
		1	0	1	2
		1	1	1	1
20,8	48,0	1	1	1	1

24,6	51,0	1	1	1	2
		2		1	
		1	1	1	1
		1	1	0	
		2		1	
		2		1	
25,5	50,0	1	1	1	1
32,8	46,0	2		1	
19,0	45,0	1	1	1	3
		1	1	1	1
28,5		1	0	1	2
		1	1	1	1
23,0	58,0	1	0	1	2
		1	1	1	1
		1	1	1	3
		1	0	1	1
26,0	48,0	1	1	1	3
34,0	42,0	1	1	1	1
19,0	49,0	1	0	1	1
		1	1	1	1
20,0		1	1	1	1
		2		1	
		2		1	
		1	1	0	
29,0	51,0	1	0	1	2
		1	0	1	2
26,0	50,0	1	0	0	
		1	1	1	1
		1	1	1	1
		2		1	
		1	1	1	1
		1	1	1	1
		2		1	
		2		1	
		1	1	1	1
		1	1	1	1
32,0	41,0	2		1	
17,6	51,0	1	1	1	3
21,0	48,0	1	1	1	1
		2		1	
		1	1	1	1
		2		1	
		2		1	
		2		1	
		1	0	1	3
		1	1	1	1
		1	1	1	1
32,0	56,0	2		1	
29,0		2		1	
16,0	46,0	1	1	1	3
		1	1	1	3
		1	0	0	
20,2	47,0	2		1	
26,6	44,0	1	1	1	3
38,7	57,0	1	1	1	1
24,0	52,0	2		1	

27,0	51,0	2		1	
		1	0	1	1
		1	0	1	1
29,0		1	0	0	
		2		1	
		1	0	1	1
		1	1	1	1
		1	0	1	1
		1	1	1	3
		1	0	0	
		1	1	1	1
27,0	87,0	1	0	0	
29,0	48,0	1	0	1	1
		1	0	1	3
		1	1	1	1
		1	0	1	3
		1	0	1	2
		2		1	
		2		1	
23,0		1	1	1	1
		1	0	0	
		1	1	1	1
		1	0	1	1
		1	1	0	
		1	1	1	1
		2		1	
		1	1	1	1
		1	1	1	1
		2		1	
		1	1	1	1
		1	1	1	1
29,0		1	0	1	2
34,0	42,5	1	1	1	3
		1	1	1	2
		1	1	1	1
		1	0	1	1
28,0		1	0	1	1
23,0	45,0	1	0	0	
		1	1	1	1
27,0		1	1	1	3
		1	1	1	3
		1	1	1	1
23,0	42,5	1	0	1	3
		1	1	1	3
		1	1	1	1
		1	0	1	1
22,0		1	1	1	1
		1	0	1	3
		1	1	1	3
		2		1	
21,9		1	1	1	1
32,8	55,0	1	1	1	3
24,4	48,5	2		1	
32,5	45,0	1	0	1	1
		1	1	1	2
		1	1	0	

		2		1	
		1	1	1	1
		2		1	
		1	1	1	1
		1	1	1	1
		1	0	1	1
		1	0	0	
		1	1	1	1
		1	1	1	1
		1	1	1	1
		2		1	
23,0		1	1	1	1
		1	1	1	2
22,7		1	1	1	1
		2		1	
22,0		1	1	0	
24,8	51,0	1	0	1	1
		1	1	1	1
		1	1	1	3
		1	0	1	1
22,0		2		1	
29,0		1	1	0	
		2		1	
		1	1	1	1
		1	1	1	1
		1	0	0	
		2		1	
		1	0	0	
		2		1	
		1	1	1	3
		1	0	1	1
		1	0	1	1
		1	1	1	1
		2		1	
		1	0	1	1
		1	1	1	1
		1	1	1	1
29,1		1	1	1	1
		1	0	1	3
		1	0	0	
		1	1	1	1
21,1		2		1	
21,3		1	1	1	1
22,5		1	1	1	3
20,3		1	0	1	1
22,4		1	1	1	1
20,0		1	1	0	
23,0		1	1	0	
23,0		1	1	1	3
16,7		1	1	0	
28,0		1	1	1	1
20,9		1	1	1	1
33,0		1	0	1	3
22,7		1	1	1	3
		1	1	1	1
24,1		1	1	0	

		1	0	1	1
17,9		1	1	1	3
27,0		1	1	1	3
24,0		1	0	1	3
23,0		1	1	1	1
29,2		1	1	1	1
36,6		1	0	1	3
		1	0	0	
24,4		1	0	1	2
29,5		1	0	1	1
		1	0	0	
		2		1	
		1	0	1	1
		2		1	
28,1		1	0	1	3
27,8		2		1	
30,8		1	0	1	1
24,9		1	1	1	1
23,9		1	1	0	
24,0		1	1	0	
		1	0	0	
		1	0	1	2
43,0		1	1	1	2
		2		1	
25,8		1	1	1	1
21,2		1	1	1	1
22,5		1	1	1	3
		1	1	1	3
23,3		1	0	1	3
18,0		1	1	1	1
28,7		1	0	1	2
		2		1	
25,9		1	0	1	1
27,0		1	0	0	
31,4		1	0	1	3
		2		1	
31,3		1	1	1	3
21,5		1	1	1	1
25,8		1	1	1	1
29,6		1	1	1	1
20,8		1	1	1	2
23,2		1	1	1	1
24,0		1	0	0	
38,5		1	1	1	3
		2		1	
24,7		1	1	1	1
27,1		1	1	1	1
29,4		1	1	0	
22,0		2		1	
31,3		1	0	1	2
23,0		1	1	1	1
21,8		1	0	1	1
28,0		1	0	1	3
19,0		1	1	1	1
29,0		1	1	1	1
		1	1	1	1

24,3		1	1	1	2
22,4		1	1	1	1
17,4		1	1	1	1

NbreTTT	MolIPP	Mol_1	Mol_2	Mol_3	Mol_4	Mol_5	PosoIPP
13	4	0	0	0	1	0	20
13	3	0	0	1	0	0	20
8	4	0	0	0	1	0	20
12	4	0	0	0	1	0	40
11	3	0	0	1	0	0	20
11	3	0	0	1	0	0	40
6	1	1	0	0	0	0	20
4	4	0	0	0	1	0	40
11	1	1	0	0	0	0	20
8	2	0	1	0	0	0	15
12	2	0	1	0	0	0	30
6	4	0	0	0	1	0	40
6	1	1	0	0	0	0	20
11	1	1	0	0	0	0	20
4	3	0	0	1	0	0	10
6	1	1	0	0	0	0	40
8	4	0	0	0	1	0	20
10	1	1	0	0	0	0	20
9	3	0	0	1	0	0	20
7	5	0	0	0	0	1	10
12	1	1	0	0	0	0	80
13	1	1	0	0	0	0	20
12	4	0	0	0	1	0	20
8	1	1	0	0	0	0	40
9	5	0	0	0	0	1	20
13	2	0	1	0	0	0	30
7	4	0	0	0	1	0	40
5	1	1	0	0	0	0	40
6	1	1	0	0	0	0	20
11	4	0	0	0	1	0	20
10	5	0	0	0	0	1	10
10	1	1	0	0	0	0	20
8	4	0	0	0	1	0	20
8	4	0	0	0	1	0	20
8	3	0	0	1	0	0	20
8	3	0	0	1	0	0	20
8	1	1	0	0	0	0	20
7	3	0	0	1	0	0	20
5	4	0	0	0	1	0	20
7	1	1	0	0	0	0	20
11	4	0	0	0	1	0	20
12	4	0	0	0	1	0	20
7	1	1	0	0	0	0	20
11	4	0	0	0	1	0	20
6	1	1	0	0	0	0	20
9	1	1	0	0	0	0	20
15	3	0	0	1	0	0	20
13	4	0	0	0	1	0	20
6	1	1	0	0	0	0	40
12	3	0	0	1	0	0	20
9	1	1	0	0	0	0	20
11	1	1	0	0	0	0	20
10	5	0	0	0	0	1	20

7	1	1	0	0	0	0	40
7	5	0	0	0	0	1	10
7	1	1	0	0	0	0	20
7	3	0	0	1	0	0	20
19	2	0	1	0	0	0	15
9	1	1	0	0	0	0	20
3	2	0	1	0	0	0	30
	1	1	0	0	0	0	40
11	1	1	0	0	0	0	20
12	5	0	0	0	0	1	20
7	1	1	0	0	0	0	20
8	5	0	0	0	0	1	10
5	4	0	0	0	1	0	20
8	4	0	0	0	1	0	40
4	4	0	0	0	1	0	20
5	3	0	0	1	0	0	10
5	3	0	0	1	0	0	20
9	4	0	0	0	1	0	20
10	3	0	0	1	0	0	10
10	3	0	0	1	0	0	20
6	3	0	0	1	0	0	20
10	3	0	0	1	0	0	20
12	1	1	0	0	0	0	40
6	3	0	0	1	0	0	20
9	1	1	0	0	0	0	20
9	4	0	0	0	1	0	20
9	1	1	0	0	0	0	20
5	3	0	0	1	0	0	20
12	1	1	0	0	0	0	20
11	4	0	0	0	1	0	20
10	4	0	0	0	1	0	20
11	4	0	0	0	1	0	40
10	4	0	0	0	1	0	20
8	4	0	0	0	1	0	20
7	3	0	0	1	0	0	20
11	3	0	0	1	0	0	20
6	1	1	0	0	0	0	20
5	2	0	1	0	0	0	30
10	1	1	0	0	0	0	20
8	1	1	0	0	0	0	40
9	4	0	0	0	1	0	40
7	1	1	0	0	0	0	20
10	4	0	0	0	1	0	20
10	2	0	1	0	0	0	30
11	4	0	0	0	1	0	20
6	4	0	0	0	1	0	40
9	1	1	0	0	0	0	20
10	1	1	0	0	0	0	20
	1	1	0	0	0	0	40
10	4	0	0	0	1	0	20
8	1	1	0	0	0	0	40
10	5	0	0	0	0	1	10
3	1	1	0	0	0	0	20
13	1	1	0	0	0	0	20
7	4	0	0	0	1	0	20
8	3	0	0	1	0	0	20

13	1	1	0	0	0	0	40
12	4	0	0	0	1	0	40
9	1	1	0	0	0	0	40
2	2	0	1	0	0	0	30
10	1	1	0	0	0	0	20
17	1	1	0	0	0	0	40
8	5	0	0	0	0	1	10
12	1	1	0	0	0	0	40
8	3	0	0	1	0	0	20
8	3	0	0	1	0	0	20
7	1	1	0	0	0	0	20
	3	0	0	1	0	0	20
12	1	1	0	0	0	0	40
8	3	0	0	1	0	0	20
8	4	0	0	0	1	0	20
15	4	0	0	0	1	0	20
11	1	1	0	0	0	0	40
9	1	1	0	0	0	0	20
6	4	0	0	0	1	0	20
10	1	1	0	0	0	0	20
10	1	1	0	0	0	0	20
9	5	0	0	0	0	1	20
8	1	1	0	0	0	0	20
8	1	1	0	0	0	0	40
4	1	1	0	0	0	0	20
11	5	0	0	0	0	1	10
5	2	0	1	0	0	0	15
10	1	1	0	0	0	0	20
11	3	0	0	1	0	0	20
4	3	0	0	1	0	0	20
5	3	0	0	1	0	0	20
9	4	0	0	0	1	0	40
8	4	0	0	0	1	0	20
6	4	0	0	0	1	0	20
9	1	1	0	0	0	0	40
7	3	0	0	1	0	0	20
13	3	0	0	1	0	0	40
4	1	1	0	0	0	0	20
10	1	1	0	0	0	0	20
9	1	1	0	0	0	0	20
8	1	1	0	0	0	0	20
9	3	0	0	1	0	0	10
7	3	0	0	1	0	0	20
6	1	1	0	0	0	0	20
9	1	1	0	0	0	0	20
6	4	0	0	0	1	0	20
5	4	0	0	0	1	0	40
5	1	1	0	0	0	0	40
7	4	0	0	0	1	0	20
8	1	1	0	0	0	0	20
7	3	0	0	1	0	0	20
5	4	0	0	0	1	0	20
11	4	0	0	0	1	0	20
7	4	0	0	0	1	0	20
6	4	0	0	0	1	0	40
14	1	1	0	0	0	0	20

13	1	1	0	0	0	0	40
5	1	1	0	0	0	0	20
8	1	1	0	0	0	0	20
8	4	0	0	0	1	0	20
8	4	0	0	0	1	0	40
7	4	0	0	0	1	0	20
12	4	0	0	0	1	0	20
4	1	1	0	0	0	0	20
7	1	1	0	0	0	0	20
9	4	0	0	0	1	0	20
10	4	0	0	0	1	0	20
8	2	0	1	0	0	0	30
5	1	1	0	0	0	0	20
	1	1	0	0	0	0	20
	1	1	0	0	0	0	20
4	1	1	0	0	0	0	20
	1	1	0	0	0	0	20
12	1	1	0	0	0	0	40
9	5	0	0	0	0	1	10
11	1	1	0	0	0	0	20
4	3	0	0	1	0	0	20
12	2	0	1	0	0	0	30
12	3	0	0	1	0	0	20
3	1	1	0	0	0	0	20
	4	0	0	0	1	0	20
7	3	0	0	1	0	0	20
5	4	0	0	0	1	0	20
12	3	0	0	1	0	0	40
13	3	0	0	1	0	0	20
6	1	1	0	0	0	0	20
9	3	0	0	1	0	0	20
7	1	1	0	0	0	0	20
8	4	0	0	0	1	0	20
14	1	1	0	0	0	0	20
9	1	1	0	0	0	0	40
6	3	0	0	1	0	0	20
9	4	0	0	0	1	0	20
8	3	0	0	1	0	0	10
12	3	0	0	1	0	0	20
8	2	0	1	0	0	0	15
9	2	0	1	0	0	0	30
10	1	1	0	0	0	0	20
7	1	1	0	0	0	0	20
12	1	1	0	0	0	0	20
8	3	0	0	1	0	0	20
13	1	1	0	0	0	0	20
11	1	1	0	0	0	0	20
4	4	0	0	0	1	0	20
10	1	1	0	0	0	0	20
5	1	1	0	0	0	0	40
10	1	1	0	0	0	0	40
7	3	0	0	1	0	0	20
8	4	0	0	0	1	0	20
8	1	1	0	0	0	0	10
8	3	0	0	1	0	0	10
13	3	0	0	1	0	0	20

5	1	1	0	0	0	0	20
	4	0	0	0	1	0	20
	3	0	0	1	0	0	10
10	1	1	0	0	0	0	20
9	1	1	0	0	0	0	40
10	4	0	0	0	1	0	40
9	5	0	0	0	0	1	10
	4	0	0	0	1	0	40
14	4	0	0	0	1	0	40
7	4	0	0	0	1	0	20
7	3	0	0	1	0	0	10
10	5	0	0	0	0	1	20
3	1	1	0	0	0	0	40
9	1	1	0	0	0	0	20
5	2	0	1	0	0	0	15
9	3	0	0	1	0	0	20
	4	0	0	0	1	0	20
8	5	0	0	0	0	1	10
12	5	0	0	0	0	1	20
9	1	1	0	0	0	0	40
10	1	1	0	0	0	0	40
10	1	1	0	0	0	0	40
5	1	1	0	0	0	0	20
8	1	1	0	0	0	0	20
14	1	1	0	0	0	0	20
3	1	1	0	0	0	0	40
13	1	1	0	0	0	0	20
15	1	1	0	0	0	0	20
9	1	1	0	0	0	0	40
10	4	0	0	0	1	0	20
8	1	1	0	0	0	0	20
7	1	1	0	0	0	0	20
12	2	0	1	0	0	0	30
11	1	1	0	0	0	0	20
10	1	1	0	0	0	0	20
7	1	1	0	0	0	0	40
8	1	1	0	0	0	0	20
10	1	1	0	0	0	0	40
6	4	0	0	0	1	0	20
12	1	1	0	0	0	0	20
14	4	0	0	0	1	0	20
12	4	0	0	0	1	0	20
7	1	1	0	0	0	0	20
10	4	0	0	0	1	0	20
12	5	0	0	0	0	1	20
6	1	1	0	0	0	0	20
8	2	0	1	0	0	0	30
8	1	1	0	0	0	0	40
7	1	1	0	0	0	0	20
9	1	1	0	0	0	0	20
14	1	1	0	0	0	0	20
4	3	0	0	1	0	0	20
10	1	1	0	0	0	0	20
6	1	1	0	0	0	0	20
10	4	0	0	0	1	0	20
10	3	0	0	1	0	0	20

8	2	0	1	0	0	0	30
8	5	0	0	0	0	1	10
6	1	1	0	0	0	0	20

DoseIPP	HeureIPP	DébutIPP	IPPConforme	IPPAppIndic
1	2	3	0	
2	3	3	0	
1	3	1	0	
2	3	4	0	
1	1	4	0	
2	1	4	0	
1	1	3	0	
2	3	1	1	2
1	1	4	0	
1	1	4	0	
2	3	3	1	1
2	3	2	0	
1	1	3	1	1
1	3	3	0	
1	1	3	1	1
2	3	3	1	1
1	3	4	0	
1	3	3	0	
2	3	3	0	
1	1	2	1	1
2	5	3	0	
1	3	2	0	
1	3	2	0	
2	3	3	1	3
1	3	4	0	
2	3	3	0	
2	2	1	0	
2	1	3	0	
1	1	2	0	
1	1	2	1	1
1	3	4	0	
1	1	3	0	
1	3	3	0	
1	3	4	0	
2	3	3	0	
2	3	3	0	
1	3	4	0	
2	3	3	0	
1	3	1	1	5
1	3	4	0	
1	3	2	0	
1	1	4	0	
1	3	3	1	3
1	3	2	0	
1	3	4	0	
1	3	4	0	
2	1	4	0	
1	3	4	0	
2	3	2	0	
2	3	4	0	
1	3	4	1	1
1	3	3	0	
2	1	3	0	

2	1	3	0	
1	3	4	0	
1	3	3	0	
2	3	4	0	
1	3	4	0	
1	3	4	0	
2	1	4	1	3
2	1	3	0	
1	3	3	0	
2	3	4	0	
1	3	3	0	
1	3	4	0	
1	3	4	0	
2	1	3	0	
1	3	2	0	
1	3	1	1	3
2	3	3	0	
1	1	4	0	
1	1	4	0	
2	3	1	1	3
2	3	4	0	
2	3	1	1	1
2	1	4	0	
2	3	1	0	
1	3	4	0	
1	3	4	0	
1	3	2	0	
2	3	4	0	
1	3	4	0	
1	3	4	0	
1	3	4	0	
2	3	4	0	
1	3	4	0	
1	3	2	0	
2	3	3	0	
2	1	3	1	1
1	3	4	0	
2	1	4	0	
1	3	3	0	
2	3	3	0	
2	3	1	1	2
1	3	4	0	
1	3	3	1	1
2	3	3	0	
1	3	2	0	
2	3	3	1	2
1	3	4	0	
1	3	4	0	
2	3	4	0	
1	3	3	0	
2	3	3	1	1
1	3	3	1	1
1	3	2	0	
1	3	3	0	
1	3	3	0	
2	3	4	0	

2	3	4	0	
2	1	4	0	
2	3	3	0	
2	5	2	1	2
1	3	3	0	
2	1	4	0	
1	3	4	0	
2	1	4	0	
2	3	4	0	
2	3	3	0	
1	3	2	0	
2	3	4	0	
2	1	4	0	
2	3	3	1	6
1	3	2	0	
1	3	3	0	
2	1	2	1	2
1	3	4	0	
1	3	3	0	
1	3	3	1	1
1	3	3	1	2
2	1	4	0	
1	3	3	0	
2	3	2	1	1
1	3	3	0	
1	3	3	0	
1	3	2	1	1
1	3	2	0	
2	3	1	0	
1	3	2	0	
2	1	2	0	
2	3	2	1	2
1	1	3	1	1
1	3	4	0	
2	3	3	0	
2	3	4	0	
2	5	3	0	
1	3	3	1	1
1	3	4	0	
1	3	3	1	3
1	1	4	0	
1	1	4	0	
2	3	4	0	
1	3	3	1	2
1	1	4	0	
1	3	2	0	
2	3	4	1	1
2	1	1	1	2
1	1	2	1	6
1	3	4	1	1
2	3	4	0	
1	1	3	1	1
1	3	2	0	
1	1	2	0	
2	3	3	1	2
1	3	3	1	3

2	3	4	0	
1	3	4	0	
1	3	4	0	
1	3	4	0	
2	3	4	1	2
1	3	4	0	
1	3	4	0	
1	1	2	1	1
1	3	4	0	
1	3	3	1	1
1	3	1	1	3
2	3	4	0	
1	3	4	0	
1	3	4	0	
1	3	3	0	
1	3	3	0	
1	3	2	0	
2	1	4	0	
1	3	1	1	1
1	3	4	0	
2	3	4	0	
2	3	3	1	1
2	3	2	1	3
1	3	3	1	1
1	3	2	0	
2	3	4	0	
1	1	4	0	
2	3	2	1	3
2	1	2	1	3
1	3	4	0	
2	3	2	1	1
1	3	3	0	
1	3	1	1	2
1	3	4	0	
2	3	2	1	6
2	3	4	0	
1	3	4	1	1
1	3	4	0	
2	3	4	0	
1	3	4	1	1
2	5	1	1	6
1	3	2	1	1
1	3	2	0	
1	3	3	0	
2	3	4	1	1
1	3	3	0	
1	3	1	0	
1	1	4	1	1
1	3	1	0	
2	1	1	1	1
1	3	4	0	
1	3	3	0	
1	3	4	0	
1	3	3	0	
2	3	4	0	
2	1	2	0	0

2	1	3	0	
1	1	3	0	
1	1	2	0	
1	2	2	0	
2		3	1	1
1	3	2	0	
1	1	2	1	1
1		2	0	0
1	3	3	0	
2	3	4	0	
2	3	3	1	3
1	4	2	1	1
2	3	2	0	0
1	3	3	0	
1	3	1	1	1
2	3	4	0	
1		3	1	1 et 3
1	3	4	0	
2	1	4	0	
2	3	4	0	
2	3	1	1	3
2	1	4	0	
1	3	3	0	
1	3	1	0	0
1	3	4	0	
2	3	3	0	
1	3	4	0	
1	1	4	0	
2	1	2	0	
1	3	3	0	
1	3	3	0	0
1	3	3	1	3
2	1	3	1	1
1	3	4	0	
1	1	2	0	
2	1	1	0	0
1	1	3	0	
2	3	2	1	6
1	3	3	0	0
1	3	3	0	
1	1	4	0	
1	3	3	0	
1	3	2	0	
1	3	4	0	
2	3	3	1	6
1	3	3	0	
2	1	3	0	
2	3	3	0	
1	3	2	0	
1	3	1	1	1
1		3	0	
2	3		0	
1	3	3	0	
1	3	2	0	
1	3	3	0	
2	3	3	0	

2	3	3	0	
1	3	1	1	1
1	3	3	0	

IndicApp_1	IndicApp_2	IndicApp_3	IndicApp_4	IndicApp_5
0	0	0	0	0
0	0	0	0	0
0	0	0	0	0
0	0	0	0	0
0	0	0	0	0
0	0	0	0	0
0	0	0	0	0
0	1	0	0	0
0	0	0	0	0
0	0	0	0	0
1	0	0	0	0
0	0	0	0	0
1	0	0	0	0
0	0	0	0	0
1	0	0	0	0
1	0	0	0	0
0	0	0	0	0
0	0	0	0	0
0	0	0	0	0
1	0	0	0	0
0	0	0	0	0
0	0	0	0	0
0	0	0	0	0
0	0	1	0	0
0	0	0	0	0
0	0	0	0	0
0	0	0	0	0
0	0	0	0	0
0	0	0	0	0
1	0	0	0	0
0	0	0	0	0
0	0	0	0	0
0	0	0	0	0
0	0	0	0	0
0	0	0	0	0
0	0	0	0	0
0	0	0	0	0
0	0	0	0	0
0	0	0	0	1
0	0	0	0	0
0	0	0	0	0
0	0	0	0	0
0	0	1	0	0
0	0	0	0	0
0	0	0	0	0
0	0	0	0	0
0	0	0	0	0
0	0	0	0	0
0	0	0	0	0
0	0	0	0	0
1	0	0	0	0
0	0	0	0	0
0	0	0	0	0

0	0	0	0	0
0	0	0	0	0
0	0	0	0	0
0	0	0	0	0
0	0	0	0	0
0	0	0	0	0
0	0	1	0	0
0	0	0	0	0
0	0	0	0	0
0	0	0	0	0
0	0	0	0	0
0	0	0	0	0
0	0	0	0	0
0	0	0	0	0
0	0	0	0	0
0	0	1	0	0
0	0	0	0	0
0	0	0	0	0
0	0	0	0	0
0	0	1	0	0
0	0	0	0	0
1	0	0	0	0
0	0	0	0	0
0	0	0	0	0
0	0	0	0	0
0	0	0	0	0
0	0	0	0	0
0	0	0	0	0
0	0	0	0	0
0	0	0	0	0
0	0	0	0	0
0	0	0	0	0
0	0	0	0	0
0	0	0	0	0
0	0	0	0	0
1	0	0	0	0
0	0	0	0	0
0	0	0	0	0
0	0	0	0	0
0	0	0	0	0
0	1	0	0	0
0	0	0	0	0
1	0	0	0	0
0	0	0	0	0
0	0	0	0	0
0	1	0	0	0
0	0	0	0	0
0	0	0	0	0
0	0	0	0	0
0	0	0	0	0
1	0	0	0	0
1	0	0	0	0
0	0	0	0	0
0	0	0	0	0
0	0	0	0	0
0	0	0	0	0

0	0	0	0	0
0	0	0	0	0
0	0	0	0	0
0	1	0	0	0
0	0	0	0	0
0	0	0	0	0
0	0	0	0	0
0	0	0	0	0
0	0	0	0	0
0	0	0	0	0
0	0	0	0	0
0	0	0	0	0
0	0	0	0	0
0	0	0	0	0
0	0	0	0	0
0	0	0	0	0
0	1	0	0	0
0	0	0	0	0
0	0	0	0	0
1	0	0	0	0
0	1	0	0	0
0	0	0	0	0
0	0	0	0	0
1	0	0	0	0
0	0	0	0	0
0	0	0	0	0
1	0	0	0	0
0	0	0	0	0
0	0	0	0	0
0	0	0	0	0
0	0	0	0	0
0	1	0	0	0
1	0	0	0	0
0	0	0	0	0
0	0	0	0	0
0	0	0	0	0
0	0	0	0	0
1	0	0	0	0
0	0	0	0	0
0	0	1	0	0
0	0	0	0	0
0	0	0	0	0
0	0	0	0	0
0	1	0	0	0
0	0	0	0	0
0	0	0	0	0
1	0	0	0	0
0	1	0	0	0
0	0	0	0	0
1	0	0	0	0
0	0	0	0	0
1	0	0	0	0
0	0	0	0	0
0	0	0	0	0
0	1	0	0	0
0	0	1	0	0

0	0	0	0	0
0	0	0	0	0
0	0	0	0	0
0	0	0	0	0
0	1	0	0	0
0	0	0	0	0
0	0	0	0	0
1	0	0	0	0
0	0	0	0	0
1	0	0	0	0
0	0	1	0	0
0	0	0	0	0
0	0	0	0	0
0	0	0	0	0
0	0	0	0	0
0	0	0	0	0
0	0	0	0	0
0	0	0	0	0
1	0	0	0	0
0	0	0	0	0
0	0	0	0	0
1	0	0	0	0
0	0	1	0	0
1	0	0	0	0
0	0	0	0	0
0	0	0	0	0
0	0	0	0	0
0	0	1	0	0
0	0	1	0	0
0	0	0	0	0
1	0	0	0	0
0	0	0	0	0
0	1	0	0	0
0	0	0	0	0
0	0	0	0	0
0	0	0	0	0
1	0	0	0	0
0	0	0	0	0
0	0	0	0	0
1	0	0	0	0
0	0	0	0	0
1	0	0	0	0
0	0	0	0	0
0	0	0	0	0
1	0	0	0	0
0	0	0	0	0
0	0	0	0	0
1	0	0	0	0
0	0	0	0	0
1	0	0	0	0
0	0	0	0	0
0	0	0	0	0
0	0	0	0	0
0	0	0	0	0
0	0	0	0	0
0	0	0	0	0

0	0	0	0	0
0	0	0	0	0
0	0	0	0	0
0	0	0	0	0
1	0	0	0	0
0	0	0	0	0
1	0	0	0	0
0	0	0	0	0
0	0	0	0	0
0	0	0	0	0
0	0	1	0	0
1	0	0	0	0
0	0	0	0	0
0	0	0	0	0
1	0	0	0	0
0	0	0	0	0
1	0	1	0	0
0	0	0	0	0
0	0	0	0	0
0	0	0	0	0
0	0	1	0	0
0	0	0	0	0
0	0	0	0	0
0	0	0	0	0
0	0	0	0	0
0	0	0	0	0
0	0	0	0	0
0	0	0	0	0
0	0	0	0	0
0	0	0	0	0
0	0	0	0	0
0	0	1	0	0
1	0	0	0	0
0	0	0	0	0
0	0	0	0	0
0	0	0	0	0
0	0	0	0	0
0	0	0	0	0
0	0	0	0	0
0	0	0	0	0
0	0	0	0	0
0	0	0	0	0
0	0	0	0	0
0	0	0	0	0
0	0	0	0	0
0	0	0	0	0
0	0	0	0	0
0	0	0	0	0
0	0	0	0	0
1	0	0	0	0
0	0	0	0	0
0	0	0	0	0
0	0	0	0	0
0	0	0	0	0
0	0	0	0	0
0	0	0	0	0

0	0	0	0	0
1	0	0	0	0
0	0	0	0	0

IndicApp_6	Dernière FOGD	IPPInappIndic	IndicInapp_1	IndicInapp_2
0		1 et 6	1	0
0		1 et 3 et 4	1	0
0		4 et 7	0	0
0		1	1	0
0		1 et 5	1	0
0		1 et 5 et 6	1	0
0		1 et 3 et 6	1	0
0			0	0
0		1 et 4	1	0
0		1 et4	1	0
0	3	1	0	0
0		3 et4	0	0
0	3	1	0	0
0		2 et6	0	1
0	3		0	0
0	3	4	0	0
0		4	0	0
0		3 et 4	0	0
0		1 et 3	1	0
0	3	6	0	0
0		6	0	0
0		1 et 6	1	0
0		3	0	0
0		4	0	0
0		1 et 4	1	0
0		1	1	0
0		4	0	0
0		1 et 3	1	0
0		4	0	0
0	2	7	0	0
0		1 et 3 et 7	1	0
0		1 et 3 et 4	1	0
0		1 et 6	1	0
0		1	1	0
0		1 et 4	1	0
0		3	0	0
0		4 et 5	0	0
0		4	0	0
0		4	0	0
0		1 et 4 et 5	1	0
0		1 et 7	1	0
0		1 et 6	1	
0		4	0	0
0		2 et 5	0	1
0		2	0	1
0		3 et 6	0	0
0		6	0	0
0		1	1	0
0		1 et 4	1	0
0		7	0	0
0	3	7	0	0
0		1 et 4 et 5	1	0
0		4	0	0

0		1	1	0
0		1	1	0
0		1 et 6	1	0
0		1 et 6	1	0
0		1	1	0
0		1 et 4	1	0
0			0	0
0		1 et 4	1	0
0		1 et 4	1	0
0		1 et 4	1	0
0		1	1	0
0		1 et 4 et 5	1	0
0		3 et 2 et 6	0	1
0		1 et 4	1	0
0		1	1	0
0			0	0
0		1	1	0
0		2	0	1
0		3	0	0
0			0	0
0		3	0	0
0	3	6	0	0
0		1 et 4 et 5	1	0
0		3 et 6	0	0
0		4	0	0
0		1 et 5 et 6	1	0
0		2	0	1
0		3	0	0
0		2	0	1
0		1 et 6	1	0
0		1 et 5 et 6	1	0
0		1 et7	1	0
0		1	1	0
0		1 et 9	1	0
0		1 et 6	1	0
0	3	7	0	0
0		4	0	0
0		7	0	0
0		4	0	0
0		3 et 7	0	0
0		3	0	0
0		1 et 4	1	0
0	3	6	0	0
0		2	0	1
0		3 et 5 et 6 et 9	0	0
0		4	0	0
0		1 et 7	1	0
0		6	0	0
0		1 et 3 et 6	1	0
0		3 et 5 et 6	0	0
0	3	4	0	0
0	3	4	0	0
0		1	1	0
0		1 et 4	1	0
0		1 et 7	1	0
0		4	0	0

0		6	0	0
0		1 et 9 et 6	1	0
0		2 et 4 et 7	0	1
0			0	0
0		6	0	0
0		1 et 4 et 5 et 7	1	0
0		1 et 4	1	0
0		1 et 6	1	0
0		1	1	0
0		1 et 4 et 5	1	0
0		2	0	1
0		3	0	0
0		1 et 4	1	0
1		4	0	0
0		1 et 4	1	0
0		1 et 7	1	0
0		4	0	0
0		1 et 4	1	0
0		3 et 4	0	0
0	3		0	0
0		5	0	0
0		5	0	0
0		1 et 6	1	0
0	3	3	0	0
0		1 et 5	1	0
0		1 et 4	1	0
0	3	4	0	0
0		6	0	0
0		5	0	0
0		2	0	1
0		4	0	0
0			0	0
0	3	7	0	0
0		1 et 6	1	0
0		1 et 4	1	0
0		9 et4	0	0
0		1 et 4	1	0
0	3		0	0
0		1 et 6	1	0
0		4	0	0
0		4	0	0
0		1	1	0
0		1 et 5	1	0
0		2	0	1
0		3 et 4	0	0
0		4	0	0
0	3		0	0
0		3	0	0
1		6	0	0
0	3	6	0	0
0		1 ET 4	1	0
0		4	0	0
0		4	0	0
0		6	0	0
0		2	0	1
0		1	0	0

0		1 ET 5	1	0
0		1	1	0
0		3 ET 4	0	0
0		2 ET 6	0	1
0		3	0	0
0		1	1	
0		3 ET 6	0	0
0	3		0	0
0		4	0	0
0	3	6	0	0
0		4	0	0
0		4	0	0
0		1	1	0
0		1	1	0
0		4	0	0
0		4	0	0
0		4	0	0
0		3 ET 5	0	0
0	3	4	0	0
0		5 et 6	0	0
0		1 ET 4	1	0
0	2	3	0	0
0	3	4	0	0
0	3	4	0	0
0		6 ET 3	0	0
0		1	1	0
0		2	0	1
0	3	7	0	0
0	3	4	0	0
0		1 ET 4	1	0
0	3	6	0	0
0		3 ET 7	0	0
0	1	4	0	0
0		6	0	0
1	3	6	0	0
0		5 ET 4	0	0
0	3	6	0	0
0		1 ET 4	1	0
0		6	0	0
0	3	6	0	0
1	3	4	0	0
0	3		0	0
0		9 et7	0	0
0		4 et 5	0	0
0	2	6	0	0
0		9, 1, 4	0	0
0		4 et 5	0	0
0	2	3	0	0
0		6	0	0
0	3		0	0
0		1 et 4	1	0
0		9 et **2**	0	1
0		9, 6	0	0
0		4 et 5	0	0
0		9 et 2?	0	0
0		4 et 5	0	0

0		1, 4 et 6	1	0
0		3	0	0
0		4	0	0
0		1, 6	1	0
0	2	6	0	0
0		1, 4 et 5	1	0
0	3	6	0	0
0		6	0	0
0		3, 4	0	0
0		1	1	0
0			0	0
0	2	7	0	0
0		2,5,6	0	1
0		8 4	0	0
0	2		0	0
0		9 et 4	0	0
0		4	0	0
0		3 et 6	0	0
0		9, 4	0	0
0		1, 3, 5 et 4	1	0
0		4	0	0
0		9	0	0
0		9	0	0
0		1	0	0
0		9, 7	0	0
0		9	0	0
0		5 et 6	0	0
0		3	0	0
0		1 et 4	1	0
0		1	1	0
0	3	4	0	0
0	3	7	0	0
0	2	6	0	0
0		4	0	0
0		1 et 9 et 4	1	0
0	3	5	0	0
0		8 et 4	0	0
1	3		0	0
0	3	2,5,7	0	1
0		1 et 6	1	0
0		1 et 4	1	0
0		1 , 6 et 7	1	0
0		3 et 6	0	0
0		5	0	0
1	2	7	0	0
0		6	0	0
0		6 et 7	0	0
0		1 et 2?	1	1
0		4	0	0
0	2	6	0	0
0		1 et 4	1	0
0		1 et 6	1	0
0		4	0	0
0		6	0	0
0		7	0	0
0		6	0	0

0		1	1	0
0	3	1	0	0
0		1 et 6	1	0

IndicInapp_3	IndicInapp_4	IndicInapp_5	IndicInapp_6	IndicInapp_7
0	0	0	1	0
1	1	0	0	0
0	1	0	0	1
0	0	0	0	0
0	0	1	0	0
0	0	1	1	0
1	0	0	1	0
0	0	0	0	0
0	1	0	0	0
0	1	0	0	0
0	1	0	0	0
1	0	0	0	0
0	0	0	1	0
0	0	0	1	0
0	0	0	0	0
1	0	0	1	0
0	1	0	0	0
1	4	0	0	0
0	0	0	0	0
0	0	0	1	0
0	0	0	1	0
0	0	0	1	0
1	0	0	0	0
0	1	0	0	0
0	1	0	0	0
0	0	0	0	0
0	1	0	0	0
1	0	0	0	0
0	1	0	0	0
0	0	0	0	1
1	0	0	0	1
1	1	0	0	0
0	0	0	1	0
0	0	0	0	0
0	1	0	0	0
1	0	0	0	0
0	1	1	0	0
0	1	0	0	0
1	0	0	6	0
0	1	1	0	0
0	0	0	0	1
0	0	0	1	0
0	1	0	0	0
0	0	1	0	0
0	0	0	0	0
1	0	0	1	0
0	0	0	1	0
0	0	0	0	0
0	1	0	0	0
0	0	0	0	1
0	0	0	0	1
0	1	1	0	0
0	1	0	0	0

0	0	0	0	0
0	0	0	0	0
0	0	0	1	0
0	0	0	1	0
0	0	0	0	0
0	1	0	0	0
0	0	0	0	0
0	1	0	0	0
0	1	0	0	0
0	1	0	0	0
0	0	0	0	0
0	1	1	0	0
1	0	0	1	0
0	1	0	0	0
0	0	0	0	0
0	0	0	0	0
0	0	0	0	0
0	0	0	0	0
1	0	0	0	0
0	0	0	0	0
1	0	0	0	0
0	0	0	1	0
0	1	1	0	0
1	0	0	1	0
0	1	0	0	0
0	0	1	1	0
0	0	0	0	0
1	0	0	0	0
0	0	0	0	0
0	0	0	1	0
0	0	1	1	0
0	0	0	0	1
0	0	0	0	0
0	0	0	0	0
0	0	0	0	0
0	0	0	0	1
0	1	0	0	0
0	0	0	0	1
0	1	0	0	0
1	0	0	0	1
1	0	0	0	0
0	1	0	0	0
0	0	0	1	0
0	0	0	0	0
1	0	1	1	0
0	1	0	0	0
0	0	0	0	1
0	0	0	1	0
1	0	0	1	0
1	0	1	1	0
0	1	0	0	0
0	1	0	0	0
0	0	0	0	0
0	1	0	0	0
0	0	0	0	1
0	1	0	0	0

0	0	0	1	0
0	0	0	1	0
0	1	0	0	1
0	0	0	0	0
0	0	0	1	0
0	1	1	0	1
0	1	0	0	0
0	0	0	1	0
0	0	0	0	0
0	1	1	0	0
0	0	0	0	0
1	0	0	0	0
0	1	0	0	0
0	1	0	0	0
0	1	0	0	0
0	0	0	0	1
0	1	0	0	0
0	1	0	0	0
1	1	0	0	0
0	0	0	0	0
0	0	1	0	1
0	0	1	0	0
0	0	0	1	0
1	0	0	0	0
0	0	1	0	0
0	1	0	0	0
0	1	0	0	0
0	0	0	1	0
0	0	1	0	0
0	0	0	0	0
0	1	0	0	0
0	0	0	0	0
0	0	0	0	1
0	0	0	1	0
0	1	0	0	0
0	1	0	0	0
0	1	0	0	0
0	0	0	0	0
0	0	0	1	0
0	1	1	0	0
0	1	0	0	0
0	0	0	0	0
0	0	1	0	0
1	1	0	0	0
1	1	0	0	0
0	1	0	0	0
0	0	0	0	0
1	0	0	1	0
0	0	0	1	0
0	0	0	1	0
0	1	0	0	0
0	1	0	0	0
0	1	0	0	0
0	0	0	1	0
1	0	0	0	0
0	1	0	0	0

0	0	1	0	0
0	0	0	0	0
1	1	0	0	0
0	0	0	1	0
1	0	0	0	0
1	0	0	1	0
0	0	0	0	0
0	1	0	0	0
0	0	0	1	0
0	1	0	0	0
0	1	0	0	0
0	0	0	0	0
0	0	0	0	0
0	1	0	0	0
0	1	0	0	0
0	1	0	0	0
1	0	1	0	0
0	1	0	0	0
0	0	1	1	0
0	1	0	0	0
1	0	0	0	0
0	1	0	0	0
0	1	0	0	0
1	0	0	1	0
0	0	0	0	0
0	0	0	0	0
0	0	0	0	1
0	1	0	0	0
0	1	0	0	0
0	0	0	1	0
1	0	0	0	1
0	1	0	0	0
0	0	0	1	0
0	0	0	1	0
0	1	1	0	0
0	0	0	1	0
0	1	0	0	0
0	0	0	1	0
0	0	0	1	0
0	1	0	0	0
0	0	0	0	0
0	0	0	0	1
0	1	1	0	0
0	0	0	1	0
0	1	1	0	0
0	1	1	0	0
1	0	0	0	0
0	0	0	1	0
0	0	0	0	0
0	1	0	0	0
0	0	0	0	0
0	0	0	1	0
0	1	1	0	0
0	0	0	0	0
0	1	1	0	0

0	1	0	1	0
1	0	0	0	0
0	1	0	0	0
0	0	0	6	0
0	0	0	6	0
0	1	1	0	0
0	0	0	1	0
0	0	0	6	0
1	1	0	0	0
0	0	0	0	0
0	0	0	0	0
0	0	0	0	1
0	0	1	1	0
0	1	0	0	0
0	1	0	0	0
0	1	0	0	0
0	1	0	0	0
1	0	0	1	0
0	1	0	0	0
1	1	1	0	0
0	1	0	0	0
0	0	0	1	0
0	0	0	0	0
0	1	0	0	0
0	0	0	0	1
0	0	0	0	0
0	0	1	1	0
1	1	0	0	0
0	1	0	0	0
0	0	0	1	0
0	1	0	0	0
0	0	0	0	1
0	0	0	1	0
0	1	0	0	0
0	1	0	0	0
0	1	1	0	0
0	1	0	0	0
0	0	0	0	0
0	0	1	0	1
0	0	0	1	0
0	1	0	0	0
0	0	0	1	1
1	0	0	1	0
0	0	1	0	0
0	0	0	0	1
0	0	0	1	0
0	0	0	1	1
0	0	0	0	0
0	1	0	0	0
0	0	0	1	0
0	1	0	0	0
0	0	0	1	0
0	1	0	0	0
0	0	0	1	0
0	0	0	0	1
0	0	0	1	0

0	0	0	0	0
0	0	0	0	1
0	0	0	1	0

IndicInapp_8	IndicInapp_9	Si "9-autre cause"<br>Précision
0	0	
0	0	
0	0	
0	0	
0	0	
0	0	
0	0	
0	0	
0	0	
0	0	
0	0	
0	0	
0	0	
0	0	
0	0	
0	0	
0	0	
0	0	
0	0	
0	0	
0	0	
0	0	
0	0	
0	0	
0	0	
0	0	
0	0	
0	0	
0	0	
0	0	
0	0	
0	0	
0	0	
0	0	
0	0	
0	0	
0	0	
0	0	
0	0	
0	0	
0	0	
0	0	
0	0	
0	0	
0	0	
0	0	
0	0	
0	0	
0	0	
0	0	
0	0	
0	0	
0	0	

0	0	
0	0	
0	0	
0	0	
0	0	
0	0	
0	0	
0	0	
0	0	
0	0	
0	0	
0	0	
0	0	
0	0	
0	0	
0	0	
0	0	
0	0	
0	0	
0	0	
0	0	
0	0	
0	0	
0	0	
0	0	
0	0	
0	0	
0	0	
0	0	
0	0	
0	0	
0	0	
0	0	
0	1	fragilité du cardia
0	0	
0	0	
0	0	
0	0	
0	0	
0	0	
0	0	
0	0	
0	0	
0	0	
0	1	GASTRITE
0	0	
0	0	
0	0	
0	0	
0	0	
0	0	
0	0	
0	0	
0	0	
0	0	
0	0	

0	0	
0	1	GASTRITE
0	0	
0	0	
0	0	
0	0	
0	0	
0	0	
0	0	
0	0	
0	0	
0	0	
0	0	
0	0	
0	0	
0	0	
0	0	
0	0	
0	0	
0	0	
0	0	
0	0	
0	0	
0	0	
0	0	
0	0	
0	0	
0	0	
0	0	
0	0	
0	0	
0	0	
0	0	
0	0	
0	0	
0	1	eructation
0	0	
0	0	
0	0	
0	0	
0	0	
0	0	
0	0	
0	0	
0	0	
0	0	
0	0	
0	0	
0	0	
0	0	
0	0	
0	0	
0	0	
0	0	
0	0	
0	0	

0	0	
0	0	
0	0	
0	0	
0	0	
0	0	
0	0	
0	0	
0	0	
0	0	
0	0	
0	0	
0	0	
0	0	
0	0	
0	0	
0	0	
0	0	
0	0	
0	0	
0	0	
0	0	
0	0	
0	0	
0	0	
0	0	
0	0	
0	0	
0	0	
0	0	
0	0	
0	0	
0	0	
0	0	
0	0	
0	0	
0	0	
0	0	
0	0	
0	0	
0	0	
0	1	épigastralgies récidivantes sans exploration
0	0	
0	0	
0	1	gastrite lymphocytaire
0	0	
0	0	
0	0	
0	0	
0	0	
0	1	gastrite chronique
0	1	inconnue
0	0	
0	1	Douleurs épigastriques
0	0	

0	0	
0	0	
0	0	
0	0	
0	0	
0	0	
0	0	
0	0	
0	0	hg : 11g,,
0	0	rgo éventuellement
0	0	
0	0	
0	0	0
1	0	
0	0	
0	1	gastralgies
0	0	
0	0	
0	1	Douleurs épigastriques
0	0	
0	0	
0	1	Douleurs épigastriques
0	1	pyrosis et brûlure au niveau de la gorge
0	0	
0	1	gastralgies
0	1	émorragique chez une patiente ayant des varices oesop
0	0	
0	0	
0	0	
0	0	
0	0	
0	0	
0	0	
0	0	
0	1	
0	0	
1	0	
0	0	
0	0	
0	0	
0	0	
0	0	
0	0	
0	0	
0	0	
0	0	
0	0	
0	0	
0	0	
0	0	
0	0	
0	0	
0	0	
0	0	
0	0	
0	0	

0	0	
0	0	
0	0	

CIRS_A	CIRS_B	CIRS_C	CIRS_D	CIRS_E	CIRS_F	CIRS_G
3	0	2	2	0	0	0
3	2	0	0	0	0	0
3	2	2	0	0	0	1
2	2	4	0	0	0	0
0	2	0	0	0	0	1
1	2	3	0	0	3	1
1	1	0	0	0	0	0
0	1	0	0	0	1	2
2	2	2	0	2	1	0
2	2	2	0	0	0	0
2	2	2	2	0	0	2
3	2	0	0	0	2	2
2	2	1	1	0	2	0
2	2	2	0	1	0	2
2	0	0	1	1	0	0
4	1	3	0	0	1	0
3	1	3	0	0	0	0
2	2	2	2	0	2	0
2	2	0	0	2	1	2
0	1	2	0	0	2	2
2	2	2	2	0	1	0
2	0	0	0	0	3	2
0	2	0	0	0	0	0
2	2	0	2	0	1	0
2	2	0	2	0	0	0
2	2	2	2	0	1	0
3	2	0	0	0	0	1
1	0	2	0	0	0	1
2	2	1	0	0	0	0
2	2	2	1	0	2	0
2	2	2	1	0	0	0
2	0	2	0	1	2	1
0	2	2	0	0	0	0
3	0	3	0	0	0	0
3	1	1	2	0	1	1
1	0	1	2	1	1	2
2	2	2	2	1	0	0
0	2	2	3	0	0	2
0	0	2	0	0	2	0
1	2	0	0	0	0	0
3	2	3	2	2	0	0
2	2	2	0	0	0	0
2	2	0	0	0	0	0
3	2	0	3	2	0	0
2	0	0	3	0	2	0
3	1	0	1	0	0	0
4	0	2	0	0	0	0
0	0	0	2	0	1	0
0	1	0	2	0	2	0
0	1	0	1	0	0	0
1	2	0	2	0	2	0
0	2	0	0	2	1	2
2	1	2	0	1	0	0

0	1	0	0	0	1	1
2	2	0	0	2	0	0
2	2	2	0	0	2	0
2	0	0	2	0	0	0
2	2	0	0	0	0	2
2	2	0	2	0	0	0
0	2	0	2	0	0	0
2	2	0	0	2	0	0
0	0	0	0	0	0	0
0	2	2	0	0	0	1
3	0	0	0	0	0	0
2	0	2	2	0	0	0
2	1	3	0	0	1	0
2	2	2	0	1	0	1
0	2	0	0	0	1	0
0	0	0	0	2	0	0
0	0	0	0	2	2	0
2	0	2	2	0	2	1
0	1	0	0	0	2	2
0	0	0	0	1	0	0
3	2	0	0	0	1	1
3	2	2	2	0	0	0
2	2	2	2	1	0	1
2	2	2	0	0	2	0
2	1	3	2	0	1	0
2	2	2	0	1	0	0
2	2	2	0	0	2	4
0	2	0	2	0	0	1
2	2	0	0	0	0	1
3	2	2	0	0	1	2
2	0	0	0	0	2	0
2	2	0	0	0	0	0
2	2	0	0	0	2	0
0	2	0	0	0	0	1
2	0	2	2	0	0	0
2	2	2	0	0	0	0
2	2	2	0	0	1	1
2	0	1	0	1	1	0
3	0	0	0	0	2	0
0	2	1	0	2	1	0
0	0	2	0	0	2	0
2	0	0	0	0	1	0
3	2	0	0	0	2	0
2	0	0	0	1	0	0
2	2	0	0	0	0	0
0	0	2	0	0	2	2
2	2	2	0	2	0	0
3	1	2	1	0	0	0
3	3	0	2	0	0	0
2	2	2	0	0	0	2
2	2	0	0	1	2	1
2	2	0	0	1	0	2
2	0	2	1	0	0	2
2	2	3	3	0	1	0
2	2	2	2	1	0	0
0	0	2	0	0	0	0

2	0	2	0	0	0	0
2	0	0	0	0	2	0
2	0	3	0	0	3	0
1	1	0	0	0	3	0
2	2	2	0	0	0	0
2	2	0	0	1	0	1
2	2	0	2	0	0	0
2	2	2	2	0	1	1
2	2	2	2	0	0	1
3	0	0	2	0	0	3
4	0	0	0	0	2	0
2	2	2	0	0	2	0
2	0	2	0	0	2	2
2	2	2	1	0	2	0
3	2	2	0	0	1	0
2	2	2	3	0	0	0
2	0	0	0	1	2	0
2	2	0	0	0	0	1
2	2	0	0	0	0	0
0	0	0	0	0	2	0
2	2	0	1	1	2	0
0	2	0	0	0	0	0
2	0	0	0	0	2	0
0	2	0	2	0	2	0
2	2	1	2	0	0	0
2	2	0	0	0	1	0
2	0	0	0	2	0	2
2	2	0	2	0	2	0
2	0	0	0	3	2	0
2	0	0	0	0	0	0
0	0	0	0	0	0	0
0	0	0	2	0	0	0
2	2	1	0	1	1	0
0	2	1	0	2	0	2
2	2	2	0	0	2	0
2	0	0	4	0	2	0
2	0	2	0	2	2	0
0	0	0	0	0	0	0
3	0	2	3	0	2	0
2	0	0	0	0	0	0
2	2	2	0	0	2	2
2	0	0	0	0	0	3
4	2	0	0	0	0	1
3	2	2	0	0	1	0
2	2	2	0	0	1	0
2	2	2	3	0	0	0
0	0	0	0	0	1	2
2	2	2	2	0	2	0
2	0	0	0	0	2	3
2	2	0	2	0	2	0
1	0	0	0	0	0	0
2	2	0	0	0	0	0
3	2	1	0	0	0	0
3	0	2	0	0	0	0
0	2	0	0	0	3	0
2	2	1	0	0	0	0

1	2	1	2	4	1	0
2	2	0	0	0	0	1
2	2	0	0	0	2	1
2	2	2	0	0	0	3
0	0	0	2	0	0	0
2	0	0	0	2	0	0
2	2	2	0	0	2	1
2	0	0	0	0	0	0
0	2	1	0	0	0	0
2	2	2	0	0	0	0
0	2	0	2	0	0	0
4	2	2	0	0	2	0
0	0	0	0	0	0	0
0	0	0	0	0	0	0
3	2	3	0	0	0	0
2	2	3	0	0	0	0
2	1	2	3	0	0	2
1	2	2	2	1	0	0
2	2	2	0	0	2	0
2	0	0	0	1	0	1
0	0	0	0	0	0	0
0	2	2	2	0	0	0
1	2	1	1	1	0	0
0	0	0	0	2	0	0
2	2	2	0	1	0	0
1	1	1	2	0	2	1
2	1	2	2	2	1	1
2	2	2	1	1	0	0
1	0	1	1	3	1	1
0	2	2	0	1	0	0
0	2	0	0	0	0	0
3	0	0	0	0	0	2
2	2	0	0	0	2	0
2	2	2	1	0	0	0
0	2	0	3	0	2	0
2	0	0	3	0	0	1
2	0	0	0	0	2	0
0	0	0	0	0	0	0
2	2	0	0	0	0	0
2	2	0	0	0	2	2
2	2	0	2	0	0	0
0	0	0	0	0	0	0
1	2	2	0	0	0	0
2	0	2	0	0	0	1
2	2	2	0	0	1	0
1	0	0	2	2	1	0
2	2	1	0	0	0	0
0	2	0	0	0	2	0
3	2	0	0	0	0	0
2	1	0	0	0	2	0
2	1	0	0	2	0	0
2	2	0	0	0	2	0
4	2	3	0	0	0	0
2	2	2	0	0	0	0
2	2	4	2	0	0	0
2	2	3	0	0	0	2

3	2	0	0	2	1	1
2	0	1	0	2	1	0
2	2	2	0	0	0	0
3	2	3	2	1	0	0
2	2	1	0	0	0	0
2	0	2	0	0	0	0
0	2	1	0	0	2	0
2	2	2	0	0	0	0
2	2	2	4	0	2	0
2	3	4	0	0	0	3
2	3	0	0	1	0	0
2	2	2	0	0	2	1
0	0	2	0	0	1	0
2	2	2	0	0	0	1
0	2	2	2	2	0	2
2	0	2	3	1	2	0
2	2	1	3	1	2	3
0	3	0	0	3	1	0
2	2	0	0	0	0	0
2	4	1	0	2	1	1
3	2	0	0	0	3	3
2	2	2	1	0	2	2
0	2	0	0	0	0	0
2	2	2	0	0	2	0
2	2	0	0	0	0	0
2	2	3	2	0	1	0
3	4	1	0	2	0	0
3	2	2	2	2	0	0
2	0	0	0	0	0	0
2	2	0	2	0	0	0
3	2	2	0	1	0	1
0	1	0	0	0	3	0
1	1	1	2	0	0	1
2	2	3	0	0	1	2
1	2	2	0	1	0	0
2	0	2	0	1	0	1
0	2	0	0	1	0	1
0	0	2	0	0	3	0
1	1	1	1	0	1	0
2	2	0	0	1	0	0
2	2	0	2	0	2	0
3	2	0	0	0	1	1
2	2	2	3	0	0	0
0	2	0	0	0	0	0
2	2	1	2	0	2	2
2	0	0	1	0	0	0
2	2	2	0	0	0	0
1	0	3	0	2	2	0
2	1	2	0	2	0	2
0	0	0	0	2	1	0
2	2	3	2	0	1	1
2	0	0	0	0	0	0
2	2	1	0	0	0	0
3	1	1	2	1	0	0
1	1	0	0	1	0	1
2	0	2	0	0	1	0

0	2	0	0	2	0	0
2	2	0	0	0	0	2
2	2	0	0	0	1	0

CIRS_H	CIRS_I	CIRS_J	CIRS_K	CIRS_L	CIRS_M
0	0	1	0	0	0
0	0	0	0	0	0
0	0	0	0	0	0
4	0	0	1	0	0
0	2	0	2	2	0
0	0	0	1	0	0
0	0	0	0	0	0
0	2	2	0	0	1
0	0	0	0	2	2
0	0	0	0	0	0
0	1	2	0	0	0
0	2	0	2	0	2
0	1	0	0	2	0
0	3	0	1	0	0
0	1	2	0	0	0
0	0	0	0	0	0
0	2	0	0	0	0
1	0	0	2	0	1
0	0	1	3	0	0
0	0	0	0	0	0
3	0	2	0	0	1
0	1	3	0	3	0
0	3	0	3	0	0
0	0	0	2	0	0
0	0	0	0	2	0
0	0	0	2	0	2
0	0	0	0	1	0
0	0	1	0	0	0
0	2	0	0	0	0
0	0	0	0	1	0
0	2	0	0	0	0
1	0	0	0	2	2
0	1	0	0	2	2
0	2	2	0	2	0
1	0	0	1	0	0
0	0	0	0	2	2
0	1	0	1	1	0
0	3	0	0	1	3
0	0	0	0	0	2
0	0	1	3	4	2
0	0	0	0	0	0
0	1	0	0	0	0
0	0	0	0	0	2
0	1	0	2	0	0
0	0	2	0	0	2
0	1	0	0	0	0
0	2	0	0	0	2
0	1	0	0	0	0
2	0	0	2	2	0
0	2	1	0	0	1
0	0	0	0	0	2
0	3	0	0	0	2
0	0	2	0	0	2

1	1	0	0	0	0
2	0	0	1	0	2
0	0	2	0	2	0
0	0	2	0	0	0
0	2	0	0	0	2
0	1	0	0	2	0
0	0	0	0	0	0
0	0	0	1	0	2
0	0	1	0	2	3
0	0	0	0	2	2
0	0	0	2	0	0
0	0	0	1	0	1
0	0	0	0	0	0
0	0	0	0	3	0
0	2	0	0	0	2
0	0	0	2	0	0
0	0	0	1	0	0
0	1	1	2	0	0
0	0	0	0	2	0
0	0	0	2	0	0
0	0	0	0	0	2
0	2	0	0	2	0
0	1	2	2	0	3
3	0	2	1	0	0
0	0	1	0	0	0
0	0	0	1	0	0
0	1	0	0	0	0
0	0	3	0	0	0
0	1	2	1	0	2
0	0	0	0	2	0
0	0	2	0	0	0
0	2	1	2	2	2
2	0	2	1	2	0
0	0	0	2	0	0
0	0	0	0	0	0
0	1	1	0	2	0
2	0	0	2	1	0
0	1	0	0	0	2
0	2	2	0	0	2
0	0	0	0	2	1
0	3	3	0	0	2
0	1	0	0	3	0
2	2	0	0	0	0
0	3	1	2	0	1
0	1	0	1	0	2
0	0	0	1	0	0
0	1	0	2	0	2
0	0	2	0	0	2
0	0	0	0	1	0
0	0	0	2	0	0
0	1	0	0	2	0
0	0	0	0	0	0
0	0	0	1	0	0
1	0	0	0	0	0
0	1	1	1	1	1
0	2	0	0	0	0

0	0	0	2	0	0
0	0	2	0	2	2
0	0	2	1	2	0
0	0	2	0	0	0
0	2	2	0	0	0
0	0	0	1	0	0
0	0	0	0	0	0
2	0	0	2	0	2
0	0	0	1	0	2
0	0	0	2	1	2
0	2	0	0	0	0
0	2	2	0	0	0
0	1	1	3	1	0
0	1	2	0	2	1
0	2	0	1	0	0
0	1	1	0	0	0
0	2	1	1	0	0
0	0	0	0	0	0
0	0	0	0	0	0
0	0	0	2	2	0
0	0	0	0	0	0
0	0	0	2	0	0
0	0	0	0	0	0
0	0	1	2	0	2
0	0	1	2	0	0
0	0	1	1	0	0
0	0	0	0	0	0
0	0	1	2	0	2
0	0	0	0	0	0
0	2	0	2	0	1
0	0	0	2	2	0
0	0	0	0	0	0
0	0	0	0	0	0
0	0	0	0	0	1
0	0	0	0	1	2
2	2	0	0	0	0
0	0	0	0	0	0
0	0	0	0	0	0
0	0	0	0	0	0
0	0	1	2	0	0
1	0	0	0	1	2
0	0	0	1	0	0
0	2	0	2	0	0
0	2	1	1	2	2
0	2	0	2	0	0
0	2	2	0	0	2
0	0	2	0	2	0
0	1	0	0	0	0
0	0	0	0	0	2
0	0	2	0	2	0
0	0	0	0	0	2
0	0	2	3	1	2
0	1	2	2	0	1
1	0	0	0	0	0
0	0	0	2	0	0
0	0	0	2	1	0

0	3	1	0	4	0
0	0	0	2	0	0
0	2	0	0	0	0
0	0	0	0	0	0
0	0	0	0	0	0
0	0	0	3	0	0
0	2	0	0	0	2
0	0	0	0	1	0
0	0	0	2	0	0
0	0	0	0	0	0
0	0	1	2	0	2
0	0	0	1	0	0
0	0	0	0	0	0
0	0	0	0	0	0
0	3	1	2	0	2
0	0	2	0	0	0
1	2	2	0	0	1
0	0	0	0	0	2
0	0	0	1	0	0
0	0	1	2	0	0
0	0	0	0	0	0
0	0	0	0	2	0
0	3	1	1	3	0
0	0	0	0	0	0
0	1	0	0	0	2
0	0	1	0	0	2
0	2	1	0	0	0
1	1	0	0	1	0
0	1	1	0	3	0
0	0	2	2	0	2
0	0	0	2	2	0
0	0	1	0	2	0
0	2	0	0	0	0
0	0	1	0	0	0
0	0	0	0	1	2
1	0	0	0	0	0
0	0	0	2	0	0
0	0	0	0	2	2
1	0	2	0	2	0
1	0	0	1	0	0
0	0	1	1	1	2
0	0	0	0	0	0
0	0	0	0	0	0
0	1	0	2	0	0
0	1	1	0	2	0
0	0	0	2	2	2
0	0	0	2	2	0
0	0	0	2	0	0
0	0	1	2	0	2
1	0	1	1	0	0
0	0	0	2	0	2
0	0	0	2	1	0
0	3	1	0	1	0
0	1	1	2	3	0
0	0	0	0	1	2
0	2	0	3	1	1

0	0	1	1	2	1
0	0	0	1	0	0
0	0	0	1	2	0
0	3	0	1	1	0
0	1	2	0	0	0
0	0	0	2	0	0
1	0	0	2	2	0
0	0	0	2	1	0
0	2	0	0	2	3
0	1	0	0	0	0
0	1	0	2	0	3
0	1	0	2	2	0
0	1	1	2	1	2
0	0	0	0	2	0
0	0	2	0	0	1
0	0	2	0	0	2
0	1	1	3	0	2
0	1	0	0	0	2
0	0	0	3	0	2
0	0	0	0	4	0
0	0	0	0	1	1
0	0	0	0	0	2
0	0	0	2	0	2
0	0	0	1	2	0
0	1	0	3	0	2
1	3	0	0	0	0
0	1	0	3	0	0
0	0	0	1	2	0
0	2	1	0	2	0
0	0	0	2	0	2
0	2	0	2	0	1
0	0	0	1	2	0
0	0	2	3	2	1
0	1	1	1	0	2
0	3	1	2	3	1
0	0	0	2	0	3
0	0	2	0	2	2
0	0	1	1	0	0
0	0	0	0	0	0
0	0	1	2	0	0
0	2	0	2	0	0
0	0	1	0	0	0
0	2	0	1	0	2
0	0	2	2	0	1
0	0	2	0	0	0
0	0	0	0	0	2
0	3	1	0	1	2
3	1	0	0	0	2
0	0	0	1	2	0
0	0	0	1	0	0
0	0	0	0	2	0
0	0	0	2	0	2
3	1	0	3	2	3
1	0	1	0	0	0
0	1	0	2	2	0
0	0	0	2	0	0

1	0	1	2	0	0
0	0	1	0	1	3
0	0	0	2	2	0

CIRS_N	CIRS-Total	Hb	ClairCréat
2	10	9,2	41,0
2	7	10,3	32,0
2	10	8,7	43,0
0	13	10,9	40,0
3	12	10,8	36,0
0	11	10,7	29,0
2	4	11,4	33,0
2	11	9,5	11,0
0	13	11,9	42,7
2	8	13,5	53,0
2	15	12,1	37,0
3	18	11,8	35,0
1	12	11,8	25,0
1	14	10,9	14,0
1	8	14,1	79,0
0	9	11,7	33,0
2	11	12,2	24,0
1	15	10,9	23,0
1	14	10,0	77,0
0	7	12,4	19,0
3	18	10,6	
0	14	9,5	36,0
0	8	8,5	5,0
0	9	13,6	94,0
0	8	13,6	75,0
0	13	9,5	95,0
0	7	11,8	28,0
1	6	10,8	45,0
2	9	10,8	21,0
0	10	10,1	
2	11	13,3	18,0
0	13	7,2	72,0
1	10	7,3	43,0
1	13	12,9	30,0
0	11	11,9	63,0
0	12	13,0	40,0
1	13	10,7	29,0
1	17	12,1	23,0
2	8	14,3	50,0
3	16	9,7	43,0
0	12	9,0	65,0
2	9	12,7	56,0
3	9	12,9	65,0
1	14	12,1	41,0
3	14	8,5	29,0
1	7	14,4	32,0
1	11	13,1	25,0
0	4	13,1	41,0
0	11	10,4	41,0
0	6	12,0	34,0
0	9	11,8	22,0
0	12	11,6	16,0
0	10	12,5	46,0

0	5	13,3	37,0
2	13	11,7	52,0
2	14	12,6	41,0
0	6	14,3	80,0
3	13	11,6	26,0
1	10	9,7	35,0
0	4	12,3	44,0
0	9	12,5	32,0
0	6	15,0	32,0
2	11	10,4	45,0
0	5	12,4	71,0
1	9	13,4	60,0
1	8	10,6	67,0
2	13	10,3	35,0
2	9	12,0	30,0
2	6	11,6	52,0
0	5	8,8	57,0
2	15	10,2	29,0
0	7	12,4	45,0
0	3	12,4	64,0
3	12	12,1	27,0
1	14	9,8	26,0
2	20	11,1	34,0
0	14	12,0	68,0
2	12	15,6	58,0
2	10	12,9	26,0
1	14	12,5	48,0
0	8	11,2	26,0
0	11	10,0	
0	12	9,3	
2	8	15,9	28,0
2	15	10,4	28,0
2	15	9,8	
2	7	9,0	59,0
0	6	13,0	102,0
2	12	12,3	43,0
0	13	11,6	57,0
0	8	11,5	50,0
2	13	13,0	29,0
1	10	12,5	39,0
2	14	11,4	15,0
2	9	12,5	50,0
2	13	14,2	20,0
0	10	7,6	11,0
0	8	7,6	31,0
0	7	12,3	
1	14	12,5	35,0
0	11	10,5	79,0
1	10	12,0	35,0
0	10	9,3	22,6
0	11	12,7	24,0
2	9	9,9	33,0
3	11	14,3	27,0
0	12	14,1	45,0
3	17	10,6	38,0
2	6	12,8	27,0

0	6	12,0	45,0
2	12		39,0
0	13	5,9	45,0
1	8	14,1	115,0
0	10	12,3	23,0
2	9	10,0	61,0
2	8	11,9	51,0
0	16		
0	12	12,1	79,0
0	13	10,3	73,0
2	10	7,7	33,9
0	12	10,5	35,0
0	14	10,8	67,0
1	16	8,2	49,0
0	11	11,0	24,0
0	11	12,8	22,0
0	9	9,6	30,0
2	7	12,1	30,0
2	6	17,3	35,0
2	8	12,8	53,0
1	9	11,8	43,0
2	6	11,2	43,0
2	6	12,8	
2	13	12,6	49,0
0	10	11,4	46,0
2	9	9,8	
0	6	13,2	25,0
0	13	9,9	33,0
0	7	9,9	42,0
0	7	12,0	
2	6	12,0	68,0
1	3	10,7	73,0
0	7	10,6	43,1
2	10	14,3	69,0
0	11	11,6	37,0
0	12	17,0	
0	8	14,2	75,4
2	2	12,2	49,7
0	10	12,6	61,0
0	5	11,0	51,0
0	14	13,2	103,0
0	6	12,2	
0	11	8,8	17,0
1	17	9,4	37,0
1	12	9,4	39,0
2	17	13,0	13,0
2	9	10,9	68,0
0	11	12,2	35,0
0	9	8,9	
0	12	14,2	53,0
1	4	12,7	53,0
2	14	10,1	87,0
0	12	13,2	18,0
2	8	12,9	64,4
0	7	11,4	
1	9	10,9	98,0

0	19	12,7	
0	7	8,6	
2	11	11,9	20,0
0	9	10,6	
2	4	10,8	36,0
0	7	11,0	
0	13	10,8	25,0
0	3	10,8	29,0
0	5	11,5	
0	6	11,5	47,0
0	9	11,9	
0	11	10,6	34,0
3	3	13,1	43,0
2	2	13,4	53,0
3	19	14,2	31,0
3	12	13,6	24,0
0	16	12,6	42,0
0	10	11,8	29,0
0	9	13,3	48,0
2	9	12,3	
2	2	13,4	47,0
0	8	13,4	64,0
2	16	11,2	8,0
2	4	11,7	32,0
0	10	11,5	36,0
1	12	14,3	45,0
2	16	10,3	28,0
1	12	11,3	38,0
2	15	14,7	34,0
2	13	14,0	
2	8	10,4	39,0
0	8	11,9	42,0
2	10	9,4	25,0
0	8	13,7	64,0
0	10	11,8	
2	9	13,9	34,0
2	8	11,0	65,0
0	4	12,1	33,0
0	9	13,8	36,0
0	10	11,1	
0	11	12,1	
3	3	11,4	49,8
2	7	13,1	50,3
0	8	9,9	22,0
2	13	11,5	47,0
2	14	13,3	67,0
0	9	12,5	32,0
0	6	12,6	40,0
2	12	12,2	40,0
0	8	13,4	35,0
0	9	9,1	44,0
0	9	11,7	38,0
0	14	10,9	32,0
3	16	12,1	45,0
0	13	11,8	28,2
0	16	12,0	17,0

1	15	12,0	47,0
0	7	13,4	36,0
2	11	12,7	40,0
0	16	11,6	7,0
0	8	13,6	35,0
2	8	10,6	37,0
1	11	13,2	86,0
1	10	14,8	54,0
0	19	11,1	34,0
0	13	6,9	27,0
0	12	9,7	40,0
3	17	14,6	77,0
0	10	10,9	34,5
3	12	12,7	23,0
0	13	13,5	67,0
0	14	14,3	101,0
2	23	12,4	66,0
1	11	16,6	30,0
0	9	13,4	54,0
3	18	17,2	65,0
0	13	8,2	70,1
1	14	13,2	87,0
0	6	12,7	102,8
2	13	12,7	45,0
2	12	11,8	15,0
1	15	8,7	14,0
2	16	9,1	57,0
0	14	11,4	40,0
2	9	11,4	25,9
0	10	13,2	27,0
0	14	11,0	16,0
0	7	10,2	35,0
2	16	15,2	42,0
0	15	14,1	43,9
2	18	11,9	34,0
0	11	14,9	34,4
0	10	14,3	70,0
2	9	13,2	57,0
0	5	10,2	69,0
2	10	9,3	29,9
1	13	11,2	24,0
0	8	12,8	33,0
2	16	10,3	32,0
2	9	12,4	73,0
3	16	10,5	44,0
0	5	9,7	38,0
0	13	10,3	30,0
0	14	10,9	114,0
2	14	10,4	98,0
1	5	13,3	53,1
0	13	12,6	41,1
0	6	13,6	78,6
2	19	11,2	59,2
0	10	9,9	36,2
2	11	15,4	58,4
0	7	10,6	36,0

0	8	11,8	47,2
0	11	13,7	65,0
2	11	11,2	58,8
